# Psychometric evaluation of the Positivum beliefs and perceptions scales to inform occupational rehabilitation following injury

**DOI:** 10.1371/journal.pone.0327355

**Published:** 2025-07-11

**Authors:** Dianne M. Sheppard, Ljoudmila Busija, Gabrielle May, Dorothy Frost

**Affiliations:** 1 MedHealth, Melbourne, Victoria, Australia; 2 Monash University Accident Research Centre (Adjunct), Monash University, Clayton, Victoria, Australia; The University of Manchester, UNITED KINGDOM OF GREAT BRITAIN AND NORTHERN IRELAND

## Abstract

Negative beliefs and perceptions about one’s health and work participation can act as barriers to rehabilitation and returning to work following an injury, thus increasing the risk of long-term work disability. To prevent poor work and health outcomes it is necessary to be able to effectively measure such constructs. The aim of the present study was to perform a psychometric evaluation of the Positivum^TM^: Beliefs and Perceptions scales used with individuals with a musculoskeletal injury or condition receiving occupational rehabilitation (OR) services through a workers’ compensation or motor vehicle accident insurance scheme. Exploratory factor analysis, item response theory-based analyses, internal consistency analyses, and confirmatory factor analysis were conducted on data collected from January 2020 to April 2024 from a sample of 3,352 musculoskeletal injured individuals receiving OR services through their compensation scheme. The results of the current study demonstrated the psychometric robustness of a revised 12 item Positivum: Beliefs and Perceptions scale (PBPS), with two clear multi-item factors: Employer Perceptions and Health-related Work Beliefs as well as two single-item measures (expectations about, and perceived enjoyment of, working). Identifying those with negative beliefs and perceptions about working following an injury and at risk of prolonged work disability is the first critical step toward preventing prolonged work disability.

## Introduction

The extent of the financial and human impact of work disability on society has been clearly demonstrated as a global issue. In Australia, an estimated 786,000 people with work disability received income support during the 2015/2016 financial year [1]. With the additional 6.5 million people who were accessing employer provided leave entitlements for work incapacity, this equated to a spend of $37.2 billion Australian dollars on income support over that 12 month period, funded by a combination of government authorities, private sector insurers and employers. The extent of this economic burden clearly illustrates the urgent need to focus efforts on the prevention of work disability.

To prevent long term work disability following an injury, research and best practice guidelines suggest that a focus beyond the injury, and on attainable recovery-related goals, is essential to get people back to active social participation and sustainable work *early,* before disability takes hold. The same principles apply whether the primary injury is physical or psychological, and whether work-related or not. An individual’s level of functioning is a dynamic interaction between that person’s health condition(s), environmental factors, and personal factors [2]. There is robust evidence that injury management and rehabilitation service providers need to adopt a holistic, biopsychosocial (BPS) approach, taking into account the range of personal, psychological, social, occupational and general health factors [3,4]. Targeted and evidence-based BPS assessment can identify current barriers that require attention for interventions to be successful [5]. Of particular interest are the modifiable psychosocial determinants of health [6] as they can act as significant barriers to rehabilitation if not adequately addressed.

A past review of the working population in the UK [7] concluded that non-medical barriers, including beliefs and perceptions in relation to one’s health, work ability/disability, are often more influential than the injury or condition itself in regards to work-related outcomes [8]. This perspective is consistent with the Health Belief Model (HBM), a theoretical framework that aims to explain and predict a wide range of health behaviours, including returning to work and social participation following injury. According to this model, an individual’s decision to undertake a health behaviour in response to an injury or condition is a function of that person’s beliefs on six dimensions, three of which – the *perceived severity* of the condition/ injury, *perceived benefits* of undertaking health action, and *perceived barriers* to engaging in the positive health behaviour – are the most relevant in the context of the prevention of work disability following injury. According to the HBM, an individual is more likely to undertake a particular health behaviour if they have fewer barriers to engaging in the health behaviours, believe that their condition or injury is a serious threat to their wellbeing, and also that the benefits of the health behaviour will effectively address the condition or injury and outweigh any associated costs [6].

It follows that inaccurate beliefs or a lack of appreciation of the health benefits of work can pose barriers to rehabilitation and returning to work following an injury, thus increasing the risk of long-term work disability. Similarly, perceptions regarding the extent to which one’s employer provides support for those with similar injuries or conditions, and the perceived likelihood of experiencing stigma or discrimination, can also impact decisions to return to the workplace and consequently lead to poorer health, work and longer term quality of life outcomes following injury [9]. To prevent such poor work and health outcomes, it is necessary to be able to effectively measure constructs relating to the injured person’s beliefs and perceptions about their health condition, work, and employer support in an early intervention occupational rehabilitation (OR) setting, enabling targeted service delivery (e.g., aligned health coaching) [3].

This paper describes the psychometric evaluation of the Beliefs and Perceptions scales within the Positivum ^TM^ assessment tool – a comprehensive biopsychosocial measure designed to be applied following a musculoskeletal injury in the compensable setting. The Positivum: Beliefs and Perceptions scales emerged as a result of consistent evidence that the individual needs to be positioned as central for the prevention of work disability to be successful.

The Positivum^TM^ assessment tool as a whole was designed to assess a comprehensive range of known biopsychosocial factors that can negatively impact work and health outcomes across a socioeconomically diverse workforce. This included beliefs and perceptions relating to health and work, perceptions of the employer, expectations around recovery and commencement of work, self-efficacy, psychological distress, pain management, and daily function. The Positivum Assessment was originally developed for the disability employment sector (for clients with a wide range of presentations including disability, illness, injury, and long term health conditions); however, it quickly became apparent that the assessment was more broadly relevant for individuals with limited or reduced physical function and pain conditions with a work-related goal, particularly within the workers compensation system, to inform and tailor OR service provision. A full description of the first iteration of the Positivum Assessment (for more details, please see: https://www.medhealth.com.au/solutions/positivum/) is beyond the scope for the current paper which focuses on the Beliefs and Perceptions scales.

The aim of the present study was to perform and report a psychometric evaluation of the Positivum: Beliefs and Perceptions scales (PBPS) for use with individuals with a musculoskeletal injury or condition receiving OR services through a workers’ compensation or motor vehicle accident insurance scheme. The psychometric evaluation comprised 4 main stages, including:

- Examination of dimensionality of the measure using exploratory factor analysis (EFA),
- Item analysis using item response theory-based (IRT) analyses,
- Internal consistency analyses, and
- Assessment of factorial validity and measurement equivalence using confirmatory factor analysis (CFA).

## Methods

### Positivum: Beliefs and perceptions measure

The PBPS is part of a more comprehensive assessment of BPS factors used within the OR context, particularly with those who have been injured and are receiving support through the workers compensation or personal injury compensation regulators. Based on the HBM, the PBPS measure was developed to explore an individual’s beliefs and perceptions and their potential interference with rehabilitation goals and, in particular, return to or commencement of work.

The initial iteration of the overarching Positivum assessment emerged based on:

- A literature review in 2015 conducted by Monash University that gathered evidence on psychosocial risk factors associated with poor work and health outcomes following injury (unpublished data)
- The findings and consensus decisions of an expert panel that included a state-based workers compensation representative, an occupational physician, Monash University academics with expertise in work-related disability and injury outcomes, and vocational rehabilitation specialists (6 persons in total, all professional contacts of the research team leading the development of this new measure)

A systematic review of standardised measures that were already available and validated was then conducted to determine the suitability of these measurement tools for assessing the key BPS areas of interest, and their suitability for this context. A number of existing questionnaires were short-listed, and the expert panel re-engaged to make decisions regarding their relevance. No existing validated scales were identified that comprehensively assessed an individual’s beliefs and perceptions that were relevant to the prevention of work disability. As such, individual items deemed to be of relevance were pulled from existing scales to be considered for inclusion in a new ‘fit for purpose’ measure. Existing scales from which items were drawn and adapted included The Keele StarT Back Tool [10], Readiness for Return to Work scale [11], The Survey of Pain Attitudes [12], and the Work-related Recovery Expectations Questionnaire [13]. These items were selected and modified where necessary by a working group emerging from the expert panel.

An initial pool of 22 items was assembled by the working group that included items belonging to each of three pre-determined, evidence-informed themed beliefs and perception categories relevant to the prevention of work disability. These themes and their working definitions are shown in Table 1.

**Table 1:** The three evidence-informed beliefs and perceptions themes and their original working definitions.

Themes	Working definitions
A. Health beliefs: Beliefs and perceptions relevant to your *ability/function/pain*	Your beliefs and perceptions about your condition or disability in relation to being able to work.
B. Work Beliefs: Beliefs and perceptions related to *working*	Your beliefs and perceptions about whether you will be able to keep working or secure new employment.
C. Employer Perceptions: Beliefs and perceptions related to your *employer*	The perceptions that you hold about your employer in relation to hiring and supporting people with a health condition or disability.

Some items from the initial pool of 22 items were deemed to fit within both themes A and B, such as ‘I believe that my condition will interfere with my ability to work’. In these instances, the item was allocated to the theme that it seemed to *best fit* conceptually. In addition, some redundancy was recognised which resulted in some of the items being excluded.

The final number of items was 12 across 3 scales, including 6 items for Health Beliefs (Category A), 3 items for Work Beliefs (Category B), and 4 for Employer Perceptions (Category C). One of the items was deemed relevant for *both* the Health and Work Beliefs dimensions (“It is not really safe for me to work”) and included as such in the scoring of both dimensions. An additional single item measure assessing work-related Recovery Expectations (“I am confident that I will be working/ will still be working in 3 months”) was also included in the Positivum: Beliefs and Perceptions measure. While this item was relevant to a Beliefs and Perceptions measure in this context, it stood on its own conceptually in that it did not seem to be a good fit for any one of the three beliefs and perceptions theme/ categories; past research supports that this single item is predictive of poor outcomes for those with pain-related injuries [14].

### Administration and scoring

The questionnaire was designed for multiple modes of administration. Face to face administration was preferred, with an interviewer-administration over the phone or self-administration using a web-based data collection form also available to those receiving OR services in regional or remote areas.

Each item is scored on a 5-point Likert scale where 1 = strongly agree and 5 = strongly disagree. Positively expressed items are inverse-scored, such that a higher score always reflects a more positive response.

### Participants and procedure

For the purposes of this study, de-identified data from a total of 3,352 individuals with musculoskeletal injuries were extracted for research purposes on 24^th^ May 2024 from an existing database containing information routinely collected as part of occupational rehabilitation and return to work service provision through MedHealth. Following referral by compensation and insurance regulators to MedHealth, clients routinely complete the Positivum assessment tool, including the PBPS, as part of a comprehensive initial assessment. A link is emailed to the client who completes the survey (typically during the initial face-to-face assessment in the presence of the OR consultant), the results subsequently made available for review during the next meeting with the trained allied health consultant to inform service delivery.

This study was approved by MedHealth Executive and consultation sought regarding privacy and consent from legal counsel. Individual written, signed consent is routinely obtained from clients at the beginning of service provision and before any data collection as part of best practice, usual care as per standard procedures. Completed consent forms are uploaded to and retained on a secure customer records management system. Subsequent data analyses were conducted internally on de-identified data for the purposes of refining an existing measurement tool and associated OR service provision.

For the purposes of the current study, eligibility criteria included:

- Injured individuals receiving OR services through either workers’ compensation (WC) scheme or through motor vehicle accident compulsory third party (CTP) insurance scheme
- Referral between 1st January 2020–30th April 2024
- Musculoskeletal condition as the primary injury or condition for receiving OR services
- Aged between 18 and 64 years

Note also that if an eligible individual provided responses to the Beliefs and Perceptions questions on multiple assessment occasions, only the *earliest assessment* was included in the study.

### Statistical analyses

Only 12 items measuring the three themes of Beliefs and Perceptions were included in analyses reported here. Missing data for the Beliefs and Perceptions items were minimal, with four items (10, 11, 12, and 13) each missing one response. All available data were included in analyses. Unless otherwise indicated, results of statistical tests were interpreted at 0.05 alpha level.

### Exploratory factor analysis

EFA aimed to examine the number and content of distinct dimensions present among the 12 Beliefs and Perceptions items. The measure was hypothesised to represent three dimensions, but there was uncertainty about separation of Work Beliefs and Health Beliefs dimensions, with one item initially allocated to both dimensions. The goal of EFA is to find the smallest number of underlying dimensions (factors) that account for the relationships between items, with the amount of information represented by each factor termed its eigenvalue. Three tests were used to find an optimal number of factors: number of factors with eigenvalues >1.0 (a factor carries more information than any individual item) [15], visual examination of scree plot to identify an abrupt change in the magnitude of factor eigenvalues (retaining only factors above the break) [16], and parallel analysis [17]. Parallel analysis involves simulating multiple sets of random data (200 in the present study), which have the same number of variables and participants as real data, but no underlying factors. Each random dataset is factor-analysed and the resulting eigenvalues are averaged and compared with real data. Eigenvalues from real data that exceeded those from the random data are used to indicate the number of factors to retain.

Where dimensionality criteria disagreed, all competing solutions were assessed on: (1) simple structure – each item has a substantive (>0.32, in absolute value) [18] loading on only one factor, (2) conceptually meaningful factors, and (3) adequate variance explained by the solution overall (>50%). Communalities, which capture the amount of variance explained by a given factor solution in individual items, were also considered, with values >0.2–0.50 interpreted as good and >0.50 excellent. Items that had no substantive loadings on any of the extracted factors, cross-loading items (loading on more than one factor), and items with communalities <0.20 were considered for removal, pending results of item analyses and consideration of whether their removal jeopardised content validity of measure.

Factors were extracted with Robust Weighted Least Squares (WLSMV) estimator [19] for categorial data. Crawford-Ferguson (C-F) oblique Varimax rotation [20] was used to aid factor interpretation. To assess sensitivity of the derived solution to the choice of extraction/rotation combination, analyses were repeated with restricted maximum likelihood (MLR) extraction and C-F Geomin rotation. In interpretation of each EFA solution, factor loadings adjusted for correlations between factors (pattern coefficients) were utilised.

### Item analysis

IRT analyses aimed to assess measurement properties of items within the scales identified with EFA. In the case of a theoretically multifactorial item (4 “It is not really safe for me to work”), the item would be allocated to a scale for which it had the highest loading in EFA. The Rasch [21] model was applied to summarise the relationship between the level of a trait represented by an item (item *difficulty,* sum of responses to a given item across all persons) and the level of a trait expressed by a person (person *ability,* an individual’s score across all items). The specific type of the Rasch model applied in our study was partial credit model [22]. The original form of the Rasch model [21] was developed for dichotomous items, with various extensions subsequently proposed for items with more than two response categories. Of these, the partial credit model and the rating scale model [23] have been specifically developed for Likert-type items. A major difference between the two models is their handling of category threshold locations. Thresholds represent points on the ability continuum where two adjacent response categories are equally likely to be endorsed. Under the rating scale model, all items are constrained to have the same threshold locations whereas the partial credit model allows threshold locations to vary between items. While the rating scale model tends to be computationally less intense due to fewer parameters to be estimated (i.e., only one set of thresholds is estimated across all items), constraining thresholds to be the same across all items can potentially mask the presence of items with category disordering, where a higher response category is associated with a lower level of underlying ability. On the other hand, the partial credit model allows category thresholds to be examined for each item individually. Since category ordering was of major interest to this study, we utilised partial credit model in our IRT analyses, despite it being computationally more intense than the rating scale model.

Under the Rasch partial credit model, items and persons are positioned along a continuum, with the more able individuals expected to consistently endorse higher response options on the more difficult items. In our study, lower overall scores for items (indicating that item is more difficult to disagree with) and persons captured the less positive beliefs and perceptions about work, while higher item and person scores represented the more positive end of beliefs and perceptions.

IRT analyses assessed unidimensionality of emergent scales, their targeting ability, category ordering, and overall fit of items with their intended scales. Differential item functioning (DIF) analyses were also undertaken to examine possible presence of measurement bias. Items displaying category disordering, misfit to the Rasch model, and evidence of measurement bias were earmarked for removal.

Unidimensionality of each scale was assessed through the principal component analysis of residuals (PCAR). PCAR involves factor-analysing item correlations that are unaccounted by the primary dimension. Unidimensionality is supported when the amount of variance explained by the first residual factor is small relative to the amount of variance explained by the primary factor. Since there is no agreed definition for an acceptable amount of variance in the residual factor, we compared the results of PCAR from the observed data with those derived from data simulated to fit the Rasch model. For each scale, five simulated datasets were created, with values averaged across datasets. Scales were considered unidimensional if 1) primary dimension explained >50% of variance; 2) eigenvalue of the first residual factor was < 2.0; 3) the residual factor explained approximately the same amount of variance as that from simulated data. Overall goodness of fit of a scale with the Rasch model was consistent with non-significant log-likelihood (LL) chi-square, normed chi-square (chi-square/degrees of freedom) values 2.0–5.0, and person separation index (PSI) ≥2.0, which would indicate that the scale is able to differentiate between people with high and low levels of a trait [24].

*Targeting* represents how well scale items capture the full continuum of abilities in the population of interest. When the difficulty continuum represented by the items does not match the continuum of respondents’ ability, the underlying trait is measured imprecisely, and content validity of a scale may be jeopardised. Targeting was assessed by visually examining the distribution of person abilities and item difficulties and by comparing means (M) and standard deviations (SD) of ability levels of respondents with those of scale items.

*Category ordering* was assessed to examine functioning of response categories. When response scale is functioning as intended, response categories are hierarchically ordered from lowest to highest, with each category displaying a distinct peak on trait continuum. Category disordering is inferred when higher response categories are subsumed under the lower categories and may indicate that respondents have difficulty discriminating between the response options.

*Item fit* was assessed with mean square (MS) and z-statistics. Infit (information-weighted) and outfit (outlier-sensitive) fit statistics were utilised. MS values between 0.5 to 1.5 and z-statistics −2.5 to 2.5 were considered optimal [24]. Values below this range are indicative of possible redundancies and high values characterise “noisy” items (high measurement error). High measurement error is detrimental to quality of measurement and noisy items were considered for removal. Redundancies occur when the level of a trait targeted by an item overlaps with one or more other items. While redundancies do not degrade quality of measurement, the presence of several redundant items can make a scale unnecessarily long. In the presence of item redundancies, residual correlations >0.30 between items were used to flag pairs of closely related items, with a misfitting item of the pair considered for removal.

*DIF* occurs when groups defined by characteristics such as age or gender, for example, have different scores on an item after controlling for the overall score. The presence of DIF was assessed across groups defined by age (<45 vs ≥ 45 years), sex, body part affected (back vs other; lower limb vs other; upper limb vs other); work status (currently at work vs no or unknown), and socio-economic status (bottom 50% of postcodes on the Index of Relative Socio-economic Advantage and Disadvantage vs top 50%) [25]. A combination of DIF contrast ≥0.50 logits and significant Mantel-Haenszel chi-square was interpreted as evidence of measurement bias. To reduce the probability of false positives, Bonferroni corrected alpha (0.05/number of items) was used to interpret the significance of chi-square tests.

### Internal consistency

Following EFA and item analysis, Cronbach’s α [26] and McDonald’s ω [27] were calculated for each emergent scale. Cronbach’s α represents the “true score” portion of the observed score, and despite being one of the most frequently utilised measures of internal consistency, has been shown to produced biased estimates of true reliability of a measure under certain conditions [28,29]. On the other hand, McDonald’s ω, which is derived from CFA factor loadings, tends to give a closer approximation of true reliability of a scale than α under a range of conditions. For both coefficients, values above 0.70 were regarded as acceptable [30].

Both coefficients are reported with 95% bootstrap confidence intervals (95% CI), derived from 1000 bootstrap draws. Factor loadings and item residual variances for the calculation of ω were derived from single factor CFA models for each scale, with no residual correlations between items allowed as future use of scale scores is not expected to take into account item residual correlations.

### Confirmatory factor analysis

The goals of CFA were two-fold: to assess factorial validity of Beliefs and Perceptions measure and to evaluate measurement equivalence of the scales between individuals receiving musculoskeletal OR services through a workers’ compensation scheme and those receiving the services through the motor vehicle accident insurance scheme (WC and CTP groups, respectively). The number and content of factors for CFA was informed by EFA and IRT analyses. For model identification, factor variances were made equal to those of indicator items. In interpretation of CFA models, fully standardised coefficients were used. Factorial validity of Beliefs and Perceptions measure was supported when the hypothesised CFA model showed good overall fit with the observed data. Good fit was consistent with a non-significant chi-square test, normed chi-square 2.0–5.0, comparative fit index (CFI) and Tucker-Lewis index (TLI) >0.95, and standardised root mean square residual (SRMR) <0.08 [31,32]. Additionally, root mean square error of approximation (RMSEA) values <0.06, 0.06–0.08, 0.08–0.10, and >0.10 were interpreted as good, reasonable, mediocre, and poor fit [32,33], respectively.

Fit of CFA models was also evaluated at an item level with misfit indicated by low (≤0.32) or non-significant loading on the hypothesised factor, high uniqueness (<50% of variance shared with the rest of scale items), several residual correlations >0.05 with other items, and several modification indices (MI) >10. Misfitting items were considered for removal, if conceptually appropriate.

Testing of CFA models proceeded in a hierarchy. Single-factor model(s) were tested first in each study group, followed by a multi-factor model.

In the final step of analyses, measurement equivalence of Beliefs and Perceptions scales was evaluated in multi-group CFA to determine whether scales functioned in the same way in CTP and WC populations. Measurement equivalence testing followed a step-up approach, beginning with a configural model (least constrained model; number and composition of factors equal between groups, but factor loadings and item thresholds allowed to vary), metric model (factor loadings are equal between groups), and scalar model (factor loadings and item thresholds equal between groups). Fit of progressively more restrictive models was compared using chi-square difference tests, with significant results indicating that the scales did not function in the same way in the two groups.

While the goal of CFA was to *test* fit of Beliefs and Perceptions scales, rather than to find a well-fitting CFA model, severe misfit at the level of single-factor models was undesirable, as it would also affect the fit of subsequent multi-factor and measurement equivalence models. This is due to the more complex models presenting more opportunities for the model misfit to arise. Therefore, in the presence of suboptimal fit of single-factor models and prior to testing more complex CFA models, model modifications in the form of within-factor correlated residuals were introduced (no more than one per factor) if these made sense conceptually and improved model fit. However, no cross-factor loadings or cross-factor correlated residuals were permitted.

### Sample size

IRT analyses generally require sample size of 100 + individuals [34]. EFA and CFA are considered large sample techniques, although there are currently no agreed criteria for optimal sample size. Generally, larger sample sizes are required for EFA when the underlying dimensions are not strongly distinct, or when the number of items defining factors is not high [35]. A previous Monte Carlo simulation study suggested minimal sample size of 200 to be adequate for the purposes of fitting a theoretical CFA model to the data [36]. In the present study, there was reasonable doubt about conceptual distinctness of Work Beliefs and Health Beliefs dimensions, due to a common item. Based on considerations outlined in [35] and results reported in [36] optimal sample size for the study was estimated to be 400 individuals for both EFA and CFA in each subpopulation, with minimal acceptable sample size of 200 per subpopulation [35,36]. Since conducting EFA and CFA on the same sample could adversely impact reproducibility of factorial structure of a measure in future studies, these analyses were carried out on different samples of individuals. As a result, there were four study samples in total: calibration WC, calibration CTP, holdout WC, and holdout CTP. EFA and item analysis were carried out with calibration samples while internal consistency and CFA utilised holdout samples.

### Statistical software

EFA and CFA were carried out in Mplus version 8.5 [37] and item analyses were carried out in Winsteps version 5.8.0 [24]. Parallel analysis was carried out with Monte Carlo PCA for Parallel Analysis [38]. Remaining analyses were carried out in Stata 18 [39].

## Results

### Participants

Characteristics of the study population are summarised in Table 2 and descriptive statistics for the Positivum Beliefs and Perceptions items are in Table 3. The database contained responses of 3,352 eligible individuals. Of these 2,978 were receiving OR services through the WC scheme and 374 were part of the CTP insurance scheme. All CTP clients were included in the study, with 174 randomly allocated to a calibration sample and the remaining 200 allocated to a holdout sample, as a larger sample was deemed more important for CFA. Of the WC clients, 400 each were randomly selected for calibration and holdout samples, respectively (S1 Table).

**Table 2:** Demographic and clinical characteristics of the study population.

Characteristic	Total (N = 3,352)		WC (n = 2,978)		CTP (n = 374)	
Age, median (IQR)	44	(32–54)	44 (33–54)		41 (30–52)	
Sex	**N**	**%**	**N**	**%**	**N**	**%**
Male	1,864	55.6	1,708	57.4	156	41.7
Female	1,238	36.9	1,099	36.9	139	37.2
Other/missing	250	7.0	171	5.7	79	21.1
Employment status						
Currently working	932	27.8	886	29.8	46	12.3
Not working, capacity to return to work	349	10.4	337	11.3	12	3.2
Not working, capacity unknown or unfit	1,778	53.0	1,488	50.0	290	77.5
Not applicable/missing	293	9.0	267	9.0	26	7.0
Body part						
Back or neck	880	26.3	836	28.1	44	11.8
Lower limb	708	21.1	682	22.9	26	7.0
Upper limb	1,037	30.9	1,001	33.6	36	9.6
Other or multiple locations	727	21.7	459	15.4	268	71.7
Condition						
Fracture	510	15.2	459	15.4	51	13.6
Multiple injuries	522	15.6	278	9.3	244	65.2
Back pain	348	10.4	333	11.2	15	4.0
Pain (other or multiple locations)	931	27.8	880	29.6	51	13.6
Soft tissue disorder or injury	198	5.9	197	6.6	1	0.3
Tear/sprain/rupture	778	23.2	770	25.9	8	2.1
Other	65	1.9	61	2.0	4	1.1
RSEAD						
Lowest 30%	785	23.4	706	23.7	79	21.1
Middle 30%	1,234	36.8	1,067	35.8	167	44.7
Highest 30%	1,245	37.1	1,117	37.5	128	34.2
Missing	88	2.6	88	3.0	0	0.0

Abbreviations: WC = Workers Compensation scheme, CTP = Compulsory Third Party insurance scheme, IQR = interquartile range, RSEAD = Index of Relative Socio-economic Advantage and Disadvantage.

**Table 3:** Summary of responses on beliefs and perceptions items.

Item	Response categories	WC (n = 2,978)		CTP (n = 374)	
		N	%	N	%
1. *I believe I am capable of working (R)*	Strongly agree	269	9.0	30	8.0
	Agree	445	14.9	81	21.7
	Neither agree nor disagree	700	23.5	91	24.3
	Disagree	1,006	33.8	131	35.0
	Strongly disagree	558	18.7	41	11.0
2. *I am confident that I will be working/ will still be working in 3 months (R) ¹*	Strongly agree	80	2.7	24	6.4
	Agree	164	5.5	39	10.4
	Neither agree nor disagree	653	21.9	104	27.8
	Disagree	1,141	38.3	149	39.8
	Strongly disagree	940	31.6	58	15.5
3. *I believe my health will get worse while working*	Strongly agree	222	7.5	31	8.3
	Agree	438	14.7	79	21.1
	Neither agree nor disagree	1,314	44.1	176	47.1
	Disagree	727	24.4	70	18.7
	Strongly disagree	277	9.3	18	4.8
4. *It is not really safe for me to work*	Strongly agree	268	9.0	26	7.0
	Agree	507	17.0	88	23.5
	Neither agree nor disagree	904	30.4	113	30.2
	Disagree	950	31.9	125	33.4
	Strongly disagree	349	11.7	22	5.9
5. *Employers prefer not to hire people with disabilities*	Strongly agree	355	11.9	58	15.5
	Agree	858	28.8	105	28.1
	Neither agree nor disagree	1,090	36.6	142	38.0
	Disagree	488	16.4	51	13.6
	Strongly disagree	187	6.3	18	4.8
6. *Because of my health, employers think that I am too much trouble*	Strongly agree	230	7.7	39	10.4
	Agree	631	21.2	99	26.5
	Neither agree nor disagree	1,058	35.5	145	38.8
	Disagree	764	25.7	69	18.5
	Strongly disagree	295	9.9	22	5.9
7. *I should not work in my current condition*	Strongly agree	462	15.5	45	12.0
	Agree	601	20.2	103	27.5
	Neither agree nor disagree	767	25.8	99	26.5
	Disagree	868	29.2	107	28.6
	Strongly disagree	280	9.4	20	5.4
8. *Employers worry that I will injure myself at work*	Strongly agree	234	7.9	43	11.5
	Agree	800	26.9	134	35.8
	Neither agree nor disagree	1,115	37.4	132	35.3
	Disagree	642	21.6	53	14.2
	Strongly disagree	187	6.3	12	3.2
9. *I believe that my condition interferes with my ability to work*	Strongly agree	665	22.3	101	27.0
	Agree	1,149	38.6	154	41.2
	Neither agree nor disagree	564	18.9	68	18.2
	Disagree	447	15.0	42	11.2
	Strongly disagree	153	5.1	9	2.4
10. *My condition gets in the way of me doing things I want to*	Strongly agree	1,085	36.5	157	42.0
	Agree	1,196	40.2	150	40.1
	Neither agree nor disagree	387	13.0	40	10.7
	Disagree	233	7.8	25	6.7
	Strongly disagree	76	2.6	2	0.5
11. *Employers worry that I will need too much time off work*	Strongly agree	257	8.6	44	11.8
	Agree	661	22.2	103	27.5
	Neither agree nor disagree	1,266	42.5	164	43.9
	Disagree	630	21.2	55	14.7
	Strongly disagree	163	5.5	8	2.1
12. *I am concerned about my health and can’t think about work at the moment*	Strongly agree	367	12.3	52	13.9
	Agree	550	18.5	87	23.3
	Neither agree nor disagree	788	26.5	101	27.0
	Disagree	975	32.8	108	28.9
	Strongly disagree	297	10.0	26	7.0
13. *There are things I enjoy about working/ think I would enjoy about working (R)*	Strongly agree	17	0.6	2	0.5
	Agree	32	1.1	6	1.6
	Neither agree nor disagree	372	12.5	45	12.0
	Disagree	1,630	54.8	224	59.9
	Strongly disagree	926	31.1	97	25.9

Note: (R) denotes reverse scored items; ¹ single-item measure, not included in further analyses. Abbreviations: WC = Workers Compensation scheme, CTP = Compulsory Third Party insurance scheme.

Median age of the study population was 44 years (interquartile range 32–54 years) and slightly more than half were male (55.6%). The CTP group were slightly younger (median age 41 years), less likely to be in paid employment (12.3% vs 29.8%), and had higher proportion of multiple injuries (65.2% vs 9.3%) than the WC group.

### Exploratory factor analysis

In both WC and CTP samples, three factors had eigenvalues > 1.0. Scree plots also suggested three factors. However, only two factors in each study sample had eigenvalues greater than those of random factors (S2 Table and S1 Fig).

In three-factor solution (S3 Table), items measuring Employer Perceptions emerged as a distinct factor (Factor 3). Factor 2 was represented by two Health Beliefs items (item 9 “My condition gets in the way of me doing things I want to” and item 10 “I believe that my condition interferes with my ability to work”) and explained only 10% of variance in the data. Factor 1 was represented by the remaining Health Beliefs and Work Beliefs items. Item 13 (“There are things I enjoy about working/ think I would enjoy about working”) was cross-loading on factors 1 (0.40 WC, 0.49 CTP) and 2 (0.34 WC, 0.45 CTP).

In two-factor solution (Table 4), factor 1 captured Health Beliefs and Work Beliefs items and factor 2 contained Employer Perceptions items in both samples. Also in both samples, item 13 had no substantive loadings on either factor. Three items – 9 and 10 in both samples and 12 in WC sample – were cross-loading.

**Table 4:** Results of two-factor exploratory factor analysis solution for Positivum Beliefs and Perceptions (pattern coefficients).

Item	Initial theme	C/WC sample (n = 400)			C/CTP sample (n = 174)		
		Factor		*h*²	Factor		*h*²
		1	2		1	2	
4 It is not really safe for me to work	WB&HB	**0.82**	0.06	0.67	**0.72**	0.22	0.56
1 I believe I am capable of working (R)	WB	**0.86**	−0.06	0.75	**0.82**	0.04	0.67
3 I believe my health will get worse while working	HB	**0.56**	0.20	0.35	**0.49**	0.23	0.30
7 I should not work in my current condition	HB	**0.89**	0.06	0.79	**0.94**	0.03	0.89
12 I am concerned about my health and can’t think about work at the moment	HB	**0.60**	**0.35**	0.48	**0.81**	0.16	0.67
9 I believe that my condition interferes with my ability to work	HB	**0.62**	**0.34**	0.50	**0.49**	**0.43**	0.43
10 My condition gets in the way of me doing things I want to	HB	**0.46**	**0.43**	0.39	***0.31***	**0.41**	0.27
13 There are things I enjoy about working/ think I would enjoy about working (R)	WB	0.17	0.03	0.03	0.28	−0.06	0.08
5 Employers prefer not to hire people with disabilities	EP	−0.20	**0.72**	0.55	−0.08	**0.77**	0.60
6 Because of my health, employers think that I am too much trouble	EP	−0.03	**0.89**	0.79	−0.02	**0.85**	0.72
8 Employers worry that I will injure myself at work	EP	0.14	**0.61**	0.39	0.04	**0.69**	0.47
11 Employers worry that I will need too much time off work	EP	0.08	**0.77**	0.60	0.19	**0.68**	0.50

Note: (R) indicates reverse scored items; *h*²denotes communalities. Abbreviations: C/WC = calibration sample, Workers Compensation scheme; C/CTP = calibration sample, Compulsory Third Party insurance scheme; WB = Work Beliefs, HB = Health Beliefs, EP = Employer Perceptions. Factor correlations: r = 0.39 in WC sample and r = 0.50 in CTP sample.

While neither two- nor three-factor solutions were ideal, following a team discussion, two-factor solution was deemed preferred due to its greater parsimony. The two factors were labelled Employer Perceptions and Health-related Work Beliefs. The solution explained 62.5% and 63.7% of variance in the WC and CTP samples, respectively. Apart from item 13, communalities were acceptable in both samples.

Comparable results were obtained with MLR extraction/Geomin rotation. However, three-factor solution produced a Heywood case, with an item loading >1.0 in the CTP sample (item 7), suggesting the extraction of too many factors, thus further supporting two-factor solution as being more robust mathematically.

### Item analysis

For item analysis, two scales were specified and analysed separately: Employer Perceptions (consistent with the initial theme) and Health-related Work Beliefs (Work Beliefs and Health Beliefs items combined). Although item 13 had no loadings on either of the two factors and item 10 had a slightly larger loading on Employer Perceptions factor in CTP sample, these items were analysed within the Health-related Work Beliefs scale due to a better conceptual fit.

### Employer perceptions scale

#### Unidimensionality and overall fit.

PCAR supported unidimensionality of Employer Perceptions scale in each study sample. Variance explained by the primary dimension was adequate (WC: 57.7%, eigenvalue = 5.3; CTP: 59.2%, eigenvalue 5.8). The first residual factor had eigenvalue 1.6 in both samples and this was comparable with simulated data (WC: 1.4; CTP:1.5). Percentage of variance explained by the residual factor (WC: 17.0%; CTP: 16.0%) was slightly higher than for simulated data (WC: 11.4%; CTP: 11.8%) and chi-square test was significant (both p < 0.001) indicating misfit with the Rasch model in both samples (Table 5). However, normed chi-square values were within the acceptable range (WC: 2.0; CTP: 1.9), suggesting that the misfit could at least partially be attributed to the relatively large sample sizes. PSI values were 2.2 in WC sample and 2.3 in CTP, indicating that the scale can reliably differentiate between individuals with high and low levels of employer perceptions positivity.

**Table 5:** Item fit statistics from item response theory item analyses.

Item	C/WC sample (n = 400)						C/CTP sample (n = 174)					
	Total score	Difficulty¹ (SE)	Mean square		z-statistic		Total score	Difficulty¹ (SE)	Mean square		z-statistic	
			Infit	Outfit	Infit	Outfit			Infit	Outfit	Infit	Outfit
*Employer Perceptions, overall fit*	χ²(1525)=3010.3, p < 0.001; χ²/df = 2.0; PSI = 2.2						χ²(653)=1213.9, p < 0.001; χ²/df = 1.9; PSI = 2.3					
5 Employers prefer not to hire people with disabilities	1125	0.3 (0.1)	1.2	1.2	**3.1**	**3.0**	459	0.2 (0.1)	1.1	1.0	0.2	0.6
6 Because of my health, employers think that I am too much trouble	1251	−0.4 (0.1)	0.7	0.7	**−5.0**	**−4.9**	503	−0.4 (0.1)	0.8	0.8	−2.0	−2.2
8 Employers worry that I will injure myself at work	1186	−0.1 (0.1)	1.2	1.2	2.3	2.3	477	0.0 (0.1)	1.1	1.1	0.8	0.8
11 Employers worry that I will need too much time off work	1178	0.2 (0.1)	0.9	0.9	−1.6	−1.5	480	0.3 (0.1)	1.1	1.0	0.2	0.5
*Health-related Work Beliefs, overall fit*	χ²(3137)=6411.3, p < 0.001; χ²/df = 2.0; PSI = 2.8						χ²(1362)=2698.8, p < 0.001; χ²/df = 2.0; PSI = 2.9					
1 I believe I am capable of working (R)	1375	−0.6 (0.1)	0.8	0.8	−2.5	**−2.9**	564	−0.7 (0.1)	0.8	0.8	−2.1	−2.2
3 I believe my health will get worse while working	1281	−0.3 (0.1)	1.1	1.1	1.0	1.2	504	−0.1 (0.1)	1.1	1.2	1.4	1.3
4 It is not really safe for me to work	1302	−0.2 (0.1)	0.7	0.7	**−4.3**	**−4.1**	537	−0.3 (0.1)	0.7	0.7	**−2.9**	**−2.9**
7 I should not work in my current condition	1208	0.2 (0.1)	0.6	0.6	**−6.0**	**−6.3**	511	−0.1 (0.1)	0.6	0.6	**−4.7**	**−4.8**
9 I believe that my condition interferes with my ability to work	969	1.1 (0.1)	0.8	0.8	−2.4	−2.4	402	1.1 (0.1)	0.9	0.9	−1.5	−1.1
10 My condition gets in the way of me doing things I want to	796	2.0 (0.1)	1.2	1.1	1.3	1.8	333	2.1 (0.1)	1.5	**1.8**	**4.6**	**4.1**
12 I am concerned about my health and can’t think about work at the moment	1249	0.0 (0.1)	0.9	0.9	−2.1	−1.9	516	−0.1 (0.1)	0.6	0.7	**−3.5**	**−4.1**
13 There are things I enjoy about working/ think I would enjoy about working (R)	1650	−2.2 (0.1)	**1.9**	**1.9**	**9.0**	**9.2**	713	−1.9 (0.2)	**1.8**	**1.7**	**4.9**	**5.5**

Note: Values consistent with item misfit are bolded. ¹Higer values correspond with lower levels of positivity (item is more difficult to strongly agree with). (R) indicates reverse scored items. Abbreviations: SE = Standard error, LL = log-likelihood; C/WC = calibration sample, Workers Compensation scheme; C/CTP = calibration sample, Compulsory Third Party insurance scheme.

#### Targeting.

**Fig 1:**
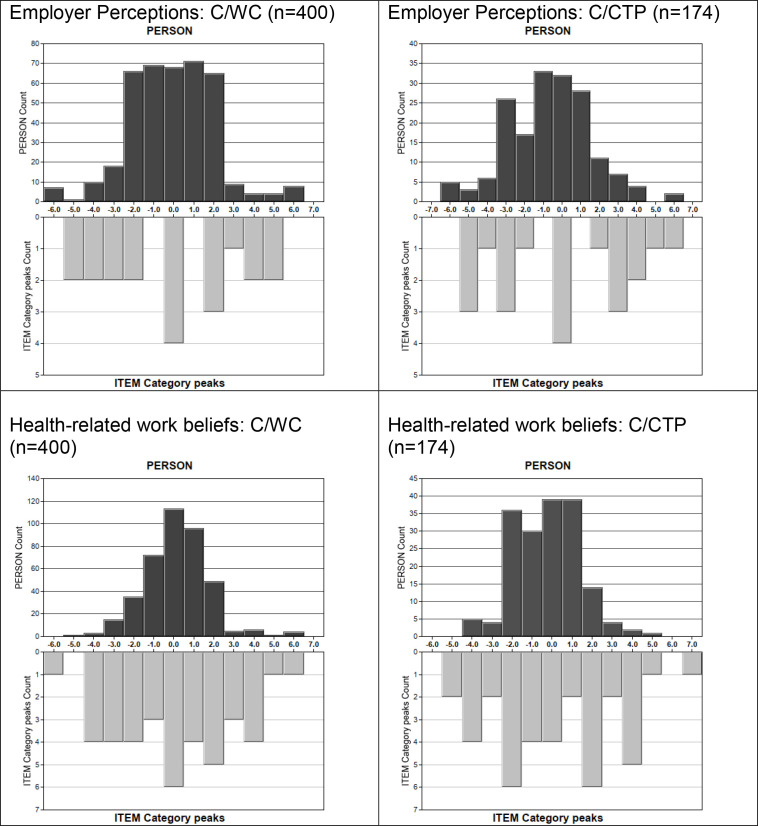
Joined distributions of person abilities and item difficulties for Beliefs and Perceptions measure. Abbreviations: C/WC = calibration sample, Workers Compensation scheme; C/CTP = calibration sample, Compulsory Third Party insurance scheme.

Mean person and item locations were reasonably close in both samples (WC: persons M = −0.1 SD = 2.1, items M = 0.0 SD = 0.3; CTP: persons M = −0.7 SD = 2.2, items M = 0.0 SD = 0.3). In both samples, levels of employer positivity represented by items were somewhat narrower than trait levels of individuals (Fig 1), indicating less measurement precision at high and low levels of employer perceptions positivity.

#### Category ordering.

**Fig 2:**
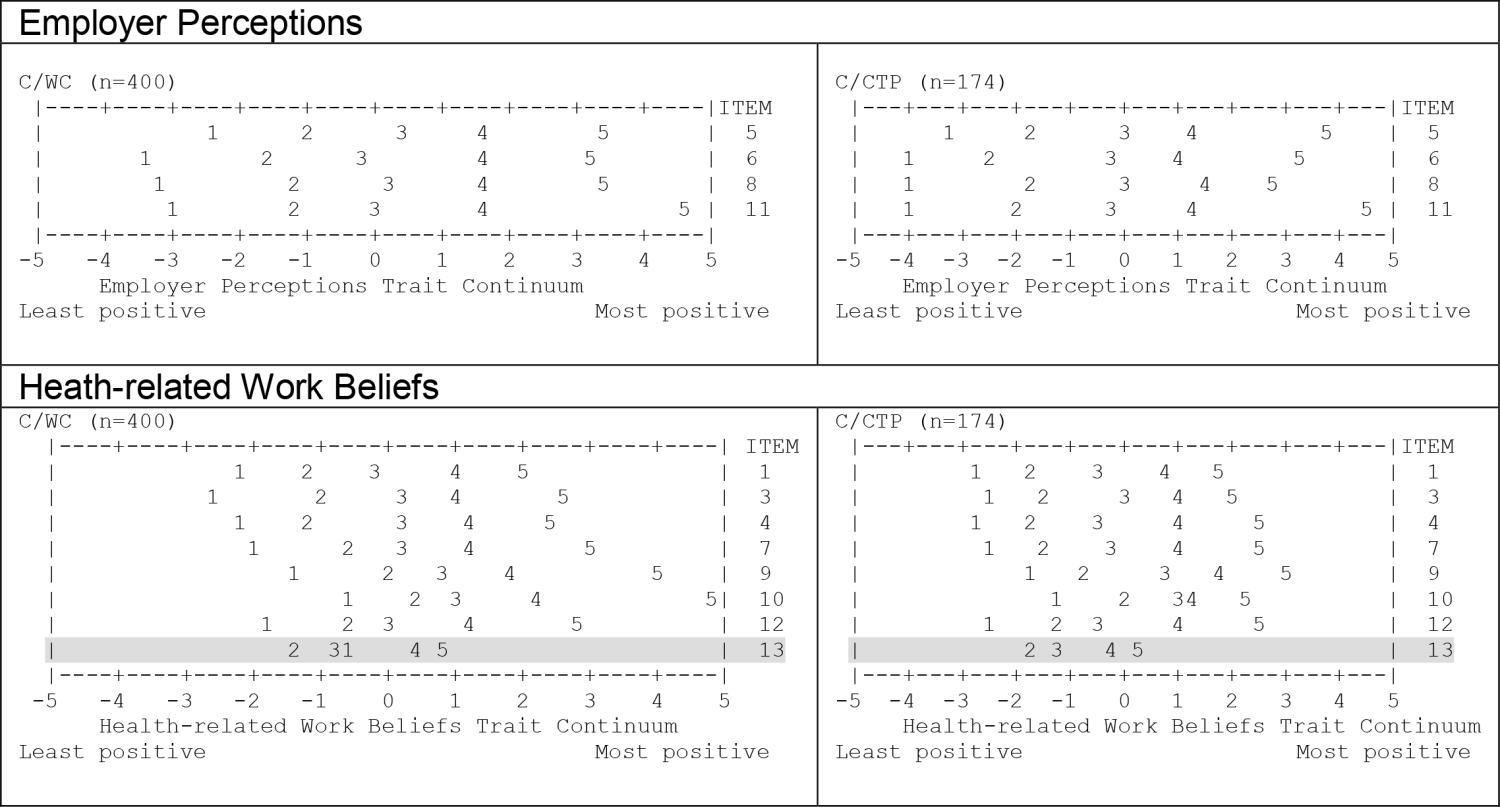
Ordering of response categories along a continuum of Beliefs and Perceptions levels. Note: Items with category disordering are highlighted. Abbreviations: C/WC = calibration sample, Workers Compensation scheme; C/CTP = calibration sample, Compulsory Third Party insurance scheme.

Positioning of response categories along the continuum of employer perceptions positivity is shown in Fig 2 (also see S2 Fig). In both samples, response categories for each item displayed a distinct peak along the trait continuum, with no evidence of category disordering.

#### Item fit.

In WC sample, according to z-statistics, item 5 (“Employers prefer not to hire people with disabilities”) had high measurement error and item 6 (“Because of my health, employers think that I am too much trouble”) was potentially redundant (Table 5). However, no clear source of item redundancy emerged, with all residual correlations in the acceptable range. In CTP sample, no misfitting items were identified.

#### Differential item functioning.

No evidence of measurement bias was found in WC sample for any of the four items. In CTP sample, item 8 (“Employers worry that I will injure myself at work”) showed evidence of measurement bias across groups defined by work status (DIF contrast = 1.0, p = 0.004). Those who were currently employed reported higher levels of disagreement with the item than individuals not in paid work or with unknown work status.

### Health-related work beliefs scale

#### Unidimensionality and overall fit.

The primary dimension explained 67.7% (eigenvalue = 16.7) and 66.5% (eigenvalue = 15.9) of variance in WC and CTP samples, respectively. The residual factor eigenvalues were within the acceptable limit (1.9 in both samples), although this was slightly higher than simulated data (WC: 1.4; CTP 1.5). Percentage of variance explained by the residual factor (WC: 7.5%; CTP: 7.8%) was lower than that from simulated data (WC: 12.1%; CTP: 12.2%), suggesting possible redundant items. Chi-square tests were significant in both samples (Table 5), while normed chi-square (2.0 in both samples) and PSI values (WC: PSI = 2.8; CTP: PSI = 2.9) were acceptable.

#### Targeting.

Fig 1 indicates that Health-related Work Beliefs scale provided good coverage of the full range of respondents’ trait levels. Average levels of health-related work beliefs positivity were also well matched by the items (WC: person M = 0.2 SD = 1.6, item M = 0.0 SD = 1.2; CTP person M = −0.3 SD = 1.1, item M = 0.0 SD = 1.6).

#### Category ordering.

Category disordering was observed for item 13 in WC sample, with response category 1 targeting higher levels of trait than categories 2 and 3 and no one endorsing response category 1 in CTP group (Fig 2; S3 Fig). Additionally, response categories 3 and 4 for item 10 in CTP sample showed redundancy, with both categories targeting the same level of underlying trait.

#### Item fit.

Item 13 showed misfit to the Rasch model on all measures of fit in both samples (Table 5). In WC sample, items 1, 4, and 7 showed evidence of redundancy on z-statistics. However, only items 9 (“I believe that my condition interferes with my ability to work”) and 10 (“My condition gets in the way of me doing things I want to”) had a substantive residual correlation (r = 0.30), identifying this pair of items as a potential source of observed redundancies. In CTP sample, z-statistics and outfit MS for item 10 indicated high amount of measurement error, while z-statistics for items 4, 7, and 12 were in the redundancy range. No residual correlations >0.30 were present.

#### Differential item functioning.

None of the items in either sample were identified as exhibiting measurement bias in either study sample.

### Item removal

No items were removed from the Employer Perceptions scale. While item 5 showed evidence of misfit on standardised measures of fit, the misfit was mild and was only present in the larger of our two samples (WC sample). Since z statistics are highly influenced by sample size, we regarded the misfit to not be detrimental to measurement properties of the scale. Additionally, item 5 had the highest difficulty score of all items in the Employer Perceptions scale and the removal of this item would further decrease the targeting ability of the scale, impacting its content validity. Hence, item 5 was retained.

In Health-related Work Beliefs scale, item 10 had high amount of measurement error and showed evidence of category disordering. As its content overlapped with that of item 9, item 10 was removed from the Beliefs and Perceptions measure. Item 13 also showed poor fit, but it was nonetheless considered important to Beliefs and Perceptions measurement as it was the only item that captured perceived enjoyment of work. It was therefore decided to leave item 13 as a single-item measure. Removal of items 10 and 13 did not impact the targeting ability of Health-related Work Beliefs scale, with PSI of 2.9 and 3.2 in WC and CTP samples, respectively. Table 6 presents the revised 12 item Positivum Beliefs and Perceptions Scales with refined definitions, and S4 Table within the supplementary information presents the revised Beliefs and Perceptions items as they pertain to the revised scales.

**Table 6:** Revised 12 item Positivum Beliefs and Perceptions Scales (PBPS) with definitions.

Revised Scales/Factors	Working definitions
A. Health-related Work beliefs: Beliefs and perceptions relevant to your *ability/function/pain that* relate to working	Your beliefs and perceptions about your condition or disability in relation to being able to work and the sustainability of your work or securing new employment.
B. Employer Perceptions: Beliefs and perceptions related to your *employer*	The perceptions that you hold about your employer in relation to hiring and supporting people with a health condition or disability.
C. Single item work expectations	Confidence that you will be working in 3 months
D. Single item perceived enjoyment of work	Having things that you (would) enjoy about working

### Internal consistency

Both Employer Perceptions and Health-related Work Beliefs scales had excellent internal consistency. Cronbach’s α for the four-item Employer Perceptions scale was 0.80 (95% CI 0.77–0.84) in WC sample (n = 400) and 0.83 (95%CI 0.79–0.88) in CTP sample (n = 200). Cronbach’s α for the six-item Health-related Work Beliefs scale was 0.90 in WC (95% CI 0.89–0.92) sample and 0.89 (95%CI 0.86–0.91) in CTP sample. McDonald’s ω for the Employer Perceptions scale was 0.84 (95% CI 0.82–0.85) and 0.87 (95% CI 0.86–0.88) in the WC and CTP samples, respectively. For the Health-related Work Beliefs scale, McDonald’s ω was 0.93 (95% CI 0.93–0.94) and 0.92 (95% CI 0.91–0.93) in the WC and CTP samples, respectively.

### Confirmatory factor analysis

#### Single-factor models.

Fit of CFA models tested in this study is summarised in Table 7. Single-factor models for Employer Perceptions scale showed a moderately good fit in both samples. CFI, TLI, and SRMR were within the acceptable range, but chi-square tests and RMSEA indices indicated misfit in both samples (Table 7, Model 1.1).

**Table 7:** Fit indices for the confirmatory factor analysis models tested in the study.

Model	χ²	df	p-value	χ²/df	Δχ²	Δdf	p-value	RMSEA	90%CI	Cfit p-value	CFI	TLI	SRMR
*Optimal range:*	*p > 0.05*			*2.0-5.0*				*<0.06*		*p > 0.05*	*>0.95*		*<0.08*
1 Employer perceptions													
1.1 Initial model													
1.1a H/WC (n = 400)	12.7	2	0.002	6.4				0.12	0.06-0.18	0.027	0.99	0.98	0.01
1.1b H/CTP (n = 200)	8.7	2	0.013	4.4				0.13	0.05-0.22	0.048	0.99	0.98	0.02
1.2 Residual correlation, items 5 and 6													
1.2a H/WC	4.9	1	0.026	4.9	8.0¹	1	0.005	0.10	0.03-0.19	0.112	1.00	0.98	0.01
1.2b H/CTP	0.0	1	0.906	0.0	7.5²	1	0.006	0.00	0.00-0.08	0.927	1.00	1.00	0.00
2 Health-related work beliefs													
2.1 Initial model													
2.1a H/WC	43.8	9	<0.001	4.9				0.10	0.07-0.13	0.003	1.00	0.99	0.02
2.1b H/CTP	70.5	9	<0.001	7.8				0.19	0.15-0.23	<0.001	0.98	0.97	0.04
2.2 Residual correlation, items 3 and 7													
2.2a H/WC	23.7	8	0.003	3.0	35.5³	1	<0.001	0.07	0.04-0.10	0.135	1.00	1.00	0.01
2.2b H/CTP	51.4	8	<0.001	6.4	28.0⁴	1	<0.001	0.17	0.12-0.21	<0.001	0.99	0.97	0.03
3 Two-factor model													
3a H/WC	137.5	32	<0.001	4.3				0.09	0.08-0.11	<0.001	0.99	0.98	0.03
3b H/CTP	101.3	32	<0.001	3.2				0.10	0.08-0.13	<0.001	0.98	0.98	0.05
4 Measurement equivalence													
4.1 Equal form	226.2	64	<0.001	3.5				0.09	0.08-0.11	<0.001	0.99	0.98	0.04
4.2 Equal factor loadings	225.8	72	<0.001	3.1	8.3⁵	8	0.402	0.08	0.07-0.10	<0.001	0.99	0.98	0.04
4.3 Equal factor loadings and item intercepts	238.5	100	<0.001	2.4	28.0⁶	28	0.464	0.07	0.06-0.08	0.004	0.99	0.99	0.04

Note: Δχ² difference between nested models (¹compared with model 1.1a, ²compared with 1.1b, ³compared with 2.1a, ⁴compared with 2.1b, ⁵compared with 4.1, ⁶ compared with 4.2). Abbreviations: H/WC = holdout sample, Workers Compensation scheme; H/CTP = holdout sample, Compulsory Third Party insurance scheme; RMSEA = root mean square error of approximation, 90%CI = 90% confidence interval for RMSEA, Cfit = test of close fit (probability of RMSEA ≤0.05), CFI = comparative fit index, TLI = Tucker-Lewis index, SRMR = standardised root mean square residual.

While the fit of Employer Perceptions scale was adequate overall, an improvement in the overall fit of a single-factor CFA model could potentially facilitate the interpretation of fit indices for the subsequent models of greater complexity, including a multi-factor CFA model and measurement equivalence testing. Hence, residual correlations and MI were inspected to identify possible model modifications that could potentially improve the fit of the current single-factor model prior to subsequent analyses.

There were no residual correlations above 0.05 in either sample, but MI indicated that correlations between residuals of items 5 and 8 or items 5 and 6 could potentially improve fit. Correlation of items 5 (“Employers prefer not to hire people with disabilities”) and 6 (“Because of my health, employers think that I am too much trouble”) was deemed conceptually defensible, as both items captured concerns with employer preferences for hiring people with disabilities. The modified model had a significantly better fit than the initial model in both samples (Table 7, Model 1.2).

Heath-related Work Beliefs scale also showed adequate overall fit to a single-factor CFA model, albeit it was also misfitting on chi-square and RMSEA tests in both samples, with a slightly better fit in WC sample (Table 7, Model 2.1). Similarly to Employer Perceptions scale, a slightly better fit could potentially facilitate the interpterion of fit indices of the more complex subsequent models. Inspection of residual correlations and MI suggested that model fit could be improved with a correlation between residuals of items 3 and 7. As both items expressed concerns about worsening health while working, the correlation was incorporated into the model, with a significant improvement in model fit (Table 7, Model 2.2), although chi-square and RMSEA remained suboptimal in CTP sample.

#### Two-factor model.

**Fig 3:**
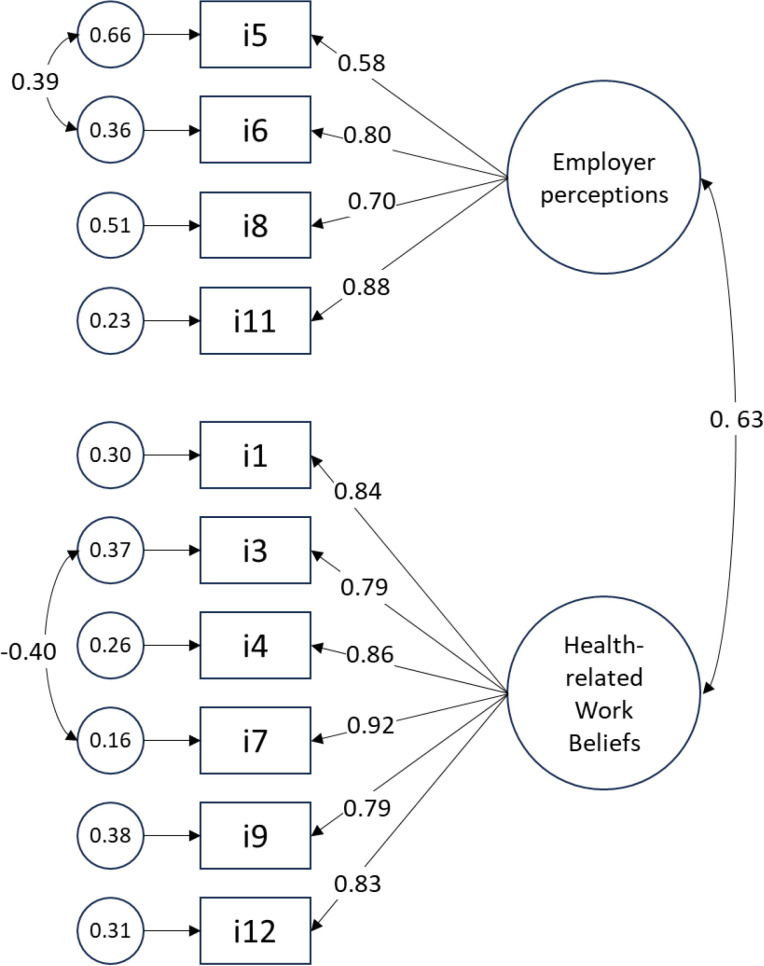
Path diagram of the two-factor model of Beliefs and Perceptions measure from the combined holdout sample. Model fit summary: χ²(32) = 195.8, p<0.001; normed χ² = 6.1; RMSEA = 0.09 (90%CI: 0.08-0.11; Probability RMSEA ≤0.05: p<0.001); CFI = 0.99, TLI = 0.98; SRMR = 0.03. Note: N = 600.

Two-factor model of Beliefs and Perceptions measure had adequate fit in both samples, supporting its factorial validity (Table 7, Model 3). While some misfit was still present on chi-square and RMSEA indices, high residual correlations and high MI were only present for cross-factor correlated errors and cross-factor loadings. Hence, no further model modifications were deemed appropriate. Path diagram of two-factor model for the combined WC and CTP holdout samples is shown in Fig 3.

#### Measurement equivalence.

The equal form solution provided adequate fit with the data (Table 7). While chi-square test was significant, RMSEA was adequate (<0.10) and the remaining indices were in the acceptable range. Constraining factor loadings to be equal across WC and CTP groups did not significantly impact fit of the model (p = 0.402) and nor did the additional constrain of equal item thresholds (p = 0.464), supporting measurement equivalence of Beliefs and Perceptions scales.

While these results provide encouraging support for the measurement equivalence of Beliefs and Perceptions scale, unequal sample sizes can potentially distort the results of multi-group CFA. To better understand the impact of group sample sizes on the results of measurement equivalence tests, we undertook post hoc simulation analyses, with five simulated datasets generated: (1) N = 200 in each group; (2) N WC = 300/ N CTP = 200 CTP; (3) N = 300 in each group; (4) N WC = 400/ N CTP = 300; (5) N = 400 in each group. Specifications for the simulated datasets were obtained from the results of the final two-factor CFA model for each group. Measurement equivalence tests were then applied to each simulated dataset.

Datasets 1–3 supported full measurement equivalence of Beliefs and Percept measure (dataset 1: metric vs. configural Δχ²(8) = 10.3, p = 0.247, scalar vs. metric Δχ²(28) = 30.7, p = 0.330; dataset 2: metric vs. configural Δχ²(8) = 7.1, p = 0.528, scalar vs. metric Δχ²(28) = 36.7, p = 0.125; dataset 3: metric vs. configural Δχ²(8) = 10.8, p = 0.212; scalar vs. metric: Δχ²(28) = 39.7, p = 0.071). Datasets 4 and 5 supported metric equivalence of Beliefs and Percept measure (metric vs. configural: dataset 4 Δχ²(8) = 11.7, p = 0.166; dataset 5 Δχ²(8) = 12.1, p = 0.145) but not its scalar equivalence (scalar vs. metric: dataset 4 Δχ²(28) = 58.6, p = 0.001; dataset 5 Δχ²(28) = 58.7, p = 0.001).

## Discussion

Work is a clear social determinant of health [40]. It follows that there is a considerable financial burden and human impact of long-term work disability which calls for more of a focus on its prevention. Consistent with the HBM, research has demonstrated that beliefs and perceptions in relation to an individual’s health, work ability/disability, are often better predictors of work-related outcomes than the injury or condition itself. Identifying those with negative beliefs and perceptions about working following an injury and at risk of prolonged work disability is the first step toward work disability prevention. As such, the present study performed a psychometric evaluation of the Positivum: Beliefs and Perceptions scales for use with individuals with a musculoskeletal injury or condition receiving OR services through a workers’ compensation or motor vehicle accident insurance scheme, with results supporting the psychometric robustness of this measurement tool. The factorial structure of the measure was clarified, with two clear factors identified. The revised 12-item measure consists of two multi-item scales (4-item Employer Perceptions and 6-item Health-related Work Beliefs) and two single-item measures (expectations about working and perceived enjoyment of working).

All but one item included in the revised version of PBPS measure showed very good fit with their respective scales in IRT analyses. The only exception was “employers prefer not to hire people with disabilities” item from the Employer Perceptions scale, with suboptimal fit on standardised indices of item fit in our larger sample (WC). However, the misfit was only mild and did not impact internal consistency of the scale, with internal consistency indices for this relatively short scale ≥0.8 – well above the minimally acceptable level of 0.70. All items included in the revised PBPS measure showed good category ordering and were free from measurement bias across diverse set of demographic and health characteristics, including age, gender, site of injury, and socio-economic status. CFA results also showed adequate fit of both Employer Perceptions and Health-related Work Beliefs scales. The results provide evidence of high internal consistency of the revised scales and their factorial validity. The study also provides strong support for metric measurement equivalence of PBPS measure in different populations receiving OP for musculoskeletal injury and moderate support for its scalar invariance. However, in a previous simulation study [41], sample sizes of 400 per group resulted in 50% to 100% of false rejections of measurement equivalence and hence it is possible that our two largest simulated datasets were overpowered. Nonetheless, metric invariance of PBPS measure across a broader range of populations and settings, as well as its scalar invariance, require further investigations, especially since one of the items of EP scale (“Employers worry that I will injure myself at work”) also displayed evidence of DIF between WC and CTP groups.

To be effectively applied in the context of work disability prevention following musculoskeletal injuries that impact work capacity, best practice recommendations prescribe early identification (within the first 3–4 weeks post-injury) of psychosocial barriers to RTW (Loisel et al., 2005; Royal Australasian College of Physicians, 2011). Unhelpful beliefs and perceptions regarding health and work, and employer support represent barriers that are amenable to change, however, while these beliefs and perceptions are held, it is likely ineffective to initiate discussions regarding returning to work [42]. In response to this the PBPS are currently implemented as part of a comprehensive BPS assessment within those initial weeks post-injury, upon referral to OR services to inform targeted and individualised service delivery for individuals looking to return to work with an approved claim.

When an individual scores poorly on one or more of the beliefs and perceptions scales evaluated by the current study, their unhelpful, negative perceptions of the employer, and/or health-related work beliefs and expectations around returning to work, can be effectively targeted by health coaching and/or therapeutic dialogue with a trained vocational rehabilitation practitioner, e.g., see [43]. This need not be limited to referrals of injured individuals *already on claim* through a worker’s compensation or motor vehicle accident scheme as per the context of the current study. Targeting these psychosocial challenges within an early intervention OR, or employer-driven injury management policies and services context, stands to improve work outcomes and prevent prolonged work disability and even prevent the need for initiating the claims process altogether. Offering such support alongside traditional OR services has been shown to be effective in building motivation and work readiness [44], with individuals moving into the vocational planning phase and subsequently graded RTW with a more positive outlook and personalised vocational goals that now seem more realistic [42].

Next steps in evaluation of the PBPS measure include examining its *stability over time* (test-retest reliability), and *responsiveness to change* or the ability to detect clinically important changes over time. Within the discipline of OR, it is often important to tie in work-related outcomes toward the end of service delivery; it would be of value to confirm that changes in the PBPS factors are predictive of positive work outcomes for individuals with a musculoskeletal injury or condition within workers’ compensation or motor vehicle accident insurance schemes. This research is already underway with plans to publish the effects of the application of the revised PBPS on work-related outcomes within the coming few years across a broader range of injury and condition types, including cancer and psychological injury. Challenges surrounding negative beliefs and perceptions about work and the employer are, of course, not limited to those with musculoskeletal and pain-related conditions. Our recently published feasibility study demonstrates the successful application of a modified Positivum assessment tailored to the cancer survivor population [45]. Currently undergoing pilot testing is another modification of the Positivum assessment specific to those with psychological injuries in response to the growing number of and expenditure on psychological claims in Australia [46].

The authors wish to acknowledge a key limitation of the study surrounding the use of data pertaining to the application of the PBPS to physical injuries specifically within the workers’ compensation and motor vehicle accident insurance context (and not more broadly). This was deliberate to ensure some degree of homogeneity across the cohort for this preliminary scale validation research. As a result, while we are confident that these results are representative of this specific context nationally, additional research will need to be undertaken to establish validity for other types of injuries and conditions within Australia, and other workforces internationally with different cultural profiles.

In conclusion, the current study demonstrated the psychometric robustness of a revised 12 item Positivum: Beliefs and Perceptions scale, with two clear multi-item factors: Employer Perceptions and Health-related Work Beliefs as well as two single-item measures (expectations about, and perceived enjoyment of, working). Identifying those with negative beliefs and perceptions about working following an injury and at risk of prolonged work disability is the first critical step toward work disability prevention. Further adaptations and application of the PBPS beyond musculoskeletal, pain related injuries and conditions is underway with versions having been developed and tailored for use with cancer survivors and those with psychological injuries in the context of returning to work.

## Supporting information

S1 Table Demographic and clinical characteristics of exploratory and confirmatory study samples. (DOCX)

S2 Table Results of dimensionality tests for exploratory factor analysis. (DOCX)

S3 Table Results of 3-factor exploratory factor analysis solution (pattern coefficients). (DOCX)

S4 Table Revised beliefs and perceptions items. (DOCX)

S1 Fig Scree plots of observed and random eigenvalues. (DOCX)

S2 Fig Category probability curves for employer perceptions items. (DOCX)

S3 Fig Category probability curves for health-related work beliefs items. (DOCX)
