# Supplementary material for: Psychometric evaluation of the Positivum beliefs and perceptions scales to inform occupational rehabilitation following injury
Source: PLoS One. 2025 Jul 11;20(7):e0327355. doi: 10.1371/journal.pone.0327355 (PMC12250564; doi:10.1371/journal.pone.0327355)
Supplement: S1 Table — (DOCX) [file pone.0327355.s001.docx]

**S1 Table**: **Demographic and clinical characteristics of exploratory and confirmatory study samples**

| Characteristic | Calibration samples | | | | |  | Holdout samples | | | | |
| --- | --- | --- | --- | --- | --- | --- | --- | --- | --- | --- | --- |
|  | WC (n=400) | |  | CTP (n=174) | |  | WC (n=400) | |  | CTP (n=200) | |
| Age, median (IQR) | 46 | (34-55) |  | 41 | (30-51) |  | 44 | (34-54) |  | 41 | (30-52) |
| Sex | **N** | **%** |  | **N** | **%** |  | **N** | **%** |  | **N** | **%** |
| Male | 222 | 55.5 |  | 67 | 38.5 |  | 212 | 53.0 |  | 89 | 44.5 |
| Female | 157 | 39.3 |  | 67 | 38.5 |  | 166 | 41.5 |  | 72 | 36.0 |
| Other/missing | 21 | 5 |  | 40 | 23.0 |  | 22 | 6.0 |  | 39 | 20.0 |
| Employment status |  |  |  |  |  |  |  |  |  |  |  |
| Currently working | 108 | 27.0 |  | 24 | 13.8 |  | 121 | 30.3 |  | 22 | 11.0 |
| Not working, capacity to return to work | 52 | 13.0 |  | 4 | 2.3 |  | 46 | 11.5 |  | 8 | 4.0 |
| Not working, capacity unknown or unfit | 206 | 51.5 |  | 137 | 78.7 |  | 193 | 48.3 |  | 153 | 76.5 |
| Not applicable/missing | 34 | 9.0 |  | 9 | 5.0 |  | 40 | 10.0 |  | 17 | 9.0 |
| Body part |  |  |  |  |  |  |  |  |  |  |  |
| Back or neck | 121 | 30.3 |  | 20 | 11.5 |  | 106 | 26.5 |  | 24 | 12.0 |
| Lower limb | 99 | 24.8 |  | 9 | 5.2 |  | 101 | 25.3 |  | 17 | 8.5 |
| Upper limb | 123 | 30.8 |  | 20 | 11.5 |  | 129 | 32.3 |  | 16 | 8.0 |
| Other or multiple locations | 57 | 14.3 |  | 125 | 71.8 |  | 64 | 16.0 |  | 143 | 71.5 |
| Condition |  |  |  |  |  |  |  |  |  |  |  |
| Fracture | 64 | 16.0 |  | 25 | 14.4 |  | 66 | 16.5 |  | 26 | 13.0 |
| Multiple injuries | 37 | 9.3 |  | 113 | 64.9 |  | 44 | 11.0 |  | 131 | 65.5 |
| Back pain | 51 | 12.8 |  | 8 | 4.6 |  | 43 | 10.8 |  | 7 | 3.5 |
| Pain (other or multiple locations) | 118 | 29.5 |  | 23 | 13.2 |  | 109 | 27.3 |  | 28 | 14.0 |
| Soft tissue disorder or injury | 22 | 5.5 |  | 1 | 0.6 |  | 26 | 6.5 |  | 0 | 0.0 |
| Tear/sprain/rupture | 99 | 24.8 |  | 3 | 1.7 |  | 105 | 26.3 |  | 5 | 2.5 |
| Other | 9 | 2.3 |  | 1 | 0.6 |  | 7 | 1.8 |  | 3 | 1.5 |
| RSEAD |  |  |  |  |  |  |  |  |  |  |  |
| Lowest 30% | 91 | 22.8 |  | 38 | 21.8 |  | 98 | 24.5 |  | 41 | 20.5 |
| Middle 30% | 140 | 35.0 |  | 72 | 41.4 |  | 142 | 35.5 |  | 95 | 47.5 |
| Highest 30% | 160 | 40.0 |  | 64 | 36.8 |  | 144 | 36.0 |  | 64 | 32.0 |
| Missing | 9 | 2.3 |  | 0 | 0.0 |  | 16 | 4.0 |  | 0 | 0.0 |

Abbreviations: WC = Workers Compensation scheme, CTP = Compulsory Third Party insurance scheme, IQR=interquartile range, RSEAD = Index of Relative Socio-economic Advantage and Disadvantage
