# Supplementary material for: Psychometric evaluation of the Positivum beliefs and perceptions scales to inform occupational rehabilitation following injury
Source: PLoS One. 2025 Jul 11;20(7):e0327355. doi: 10.1371/journal.pone.0327355 (PMC12250564; doi:10.1371/journal.pone.0327355)
Supplement: S2 Table — (DOCX) [file pone.0327355.s002.docx]

**S2 Table:** **Results of dimensionality tests for exploratory factor analysis**

| C/WC sample (N=400) | | | | | |  | C/CTP sample (N=174) | | | | | |
| --- | --- | --- | --- | --- | --- | --- | --- | --- | --- | --- | --- | --- |
| Observed eigenvalue | Random eigenvalue | SD | Decision | % of variance explained | Cumulative % |  | Observed eigenvalue | Random eigenvalue | SD | Decision | % of variance explained | Cumulative % |
| **5.76** | 1.29 | 0.04 | Retain | 48.0 | 48.0 |  | **6.19** | 1.45 | 0.07 | Retain | 51.6 | 51.6 |
| **1.73** | 1.21 | 0.03 | Retain | 14.4 | 62.5 |  | **1.46** | 1.33 | 0.05 | Retain | 12.1 | 63.7 |
| **1.12** | 1.16 | 0.03 | Discard | 9.3 | 71.8 |  | **1.09** | 1.23 | 0.04 | Discard | 9.1 | 72.8 |
| 0.65 | 1.10 | 0.02 |  | 5.4 | 77.2 |  | 0.72 | 1.16 | 0.03 |  | 6.0 | 78.8 |
| 0.57 | 1.06 | 0.02 |  | 4.8 | 82.0 |  | 0.60 | 1.08 | 0.03 |  | 5.0 | 83.8 |
| 0.54 | 1.01 | 0.02 |  | 4.5 | 86.4 |  | 0.46 | 1.01 | 0.03 |  | 3.8 | 87.6 |
| 0.41 | 0.97 | 0.02 |  | 3.5 | 89.9 |  | 0.36 | 0.95 | 0.03 |  | 3.0 | 90.6 |
| 0.33 | 0.93 | 0.02 |  | 2.7 | 92.6 |  | 0.33 | 0.89 | 0.03 |  | 2.7 | 93.4 |
| 0.26 | 0.89 | 0.02 |  | 2.2 | 94.8 |  | 0.27 | 0.83 | 0.03 |  | 2.2 | 95.6 |
| 0.25 | 0.84 | 0.02 |  | 2.1 | 96.8 |  | 0.23 | 0.76 | 0.03 |  | 1.9 | 97.5 |
| 0.22 | 0.80 | 0.03 |  | 1.8 | 98.7 |  | 0.19 | 0.69 | 0.04 |  | 1.6 | 99.1 |
| 0.16 | 0.74 | 0.03 |  | 1.3 | 100.0 |  | 0.11 | 0.61 | 0.04 |  | 0.9 | 100.0 |

Abbreviations: C/WC = calibration sample, Workers Compensation scheme; C/CTP = calibration sample, Compulsory Third Party insurance scheme; SD = standard deviation.
