# Supplementary material for: Psychometric evaluation of the Positivum beliefs and perceptions scales to inform occupational rehabilitation following injury
Source: PLoS One. 2025 Jul 11;20(7):e0327355. doi: 10.1371/journal.pone.0327355 (PMC12250564; doi:10.1371/journal.pone.0327355)
Supplement: S3 Table — (DOCX) [file pone.0327355.s003.docx]

**S3 Table**: Results of 3-factor exploratory factor analysis solution (pattern coefficients)

| Item | Label | Initial theme | C/WC sample (N=400) | | | |  | C/CTP sample (N=174) | | | |
| --- | --- | --- | --- | --- | --- | --- | --- | --- | --- | --- | --- |
|  |  |  | Factor | | | *h*² |  | Factor | | | *h*² |
|  |  |  | 1 | 2 | 3 |  |  | 1 | 2 | 3 |  |
| 4 | It is not really safe for me to work | WB&HB | **0.75** | 0.14 | 0.07 | 0.58 |  | **0.64** | 0.17 | 0.20 | 0.48 |
| 1 | I believe I am capable of working | WB | **0.82** | 0.08 | -0.02 | 0.67 |  | **0.69** | 0.27 | -0.02 | 0.54 |
| 13 | There are things I enjoy about working / think I would enjoy about working | WB | **0.40** | **-0.34** | 0.20 | 0.31 |  | **0.49** | **-0.45** | 0.17 | 0.47 |
| 3 | I believe my health will get worse while working | HB | **0.54** | 0.08 | 0.22 | 0.34 |  | **0.47** | 0.08 | 0.24 | 0.28 |
| 7 | I should not work in my current condition | HB | **0.74** | 0.28 | 0.00 | 0.62 |  | **0.83** | 0.22 | 0.01 | 0.73 |
| 12 | I am concerned about my health and can’t think about work at the moment | HB | **0.49** | 0.25 | 0.28 | 0.38 |  | **0.74** | 0.14 | 0.17 | 0.59 |
| 9 | I believe that my condition interferes with my ability to work | HB | 0.25 | **0.72** | 0.02 | 0.58 |  | 0.21 | **0.69** | 0.13 | 0.54 |
| 10 | My condition gets in the way of me doing things I want to | HB | -0.04 | **0.86** | 0.08 | 0.75 |  | 0.00 | **0.69** | 0.10 | 0.49 |
| 5 | Employers prefer not to hire people with disabilities | EP | -0.12 | -0.01 | **0.69** | 0.48 |  | -0.07 | 0.13 | **0.69** | 0.50 |
| 6 | Because of my health, employers think that I am too much trouble | EP | 0.05 | 0.00 | **0.90** | 0.81 |  | 0.02 | 0.00 | **0.87** | 0.76 |
| 8 | Employers worry that I will injure myself at work | EP | 0.04 | 0.27 | **0.49** | 0.32 |  | 0.01 | 0.16 | **0.61** | 0.40 |
| 11 | Employers worry that I will need too much time off work | EP | -0.01 | 0.27 | **0.65** | 0.49 |  | 0.11 | 0.26 | **0.57** | 0.40 |

Note: *h*²=communality. Abbreviations: C/WC = calibration sample, Workers Compensation scheme; C/CTP = calibration sample, Compulsory Third Party insurance scheme; WB = Work Beliefs, HB = Health Beliefs, EP = Employer Perceptions.
