# Supplementary material for: Psychometric evaluation of the Positivum beliefs and perceptions scales to inform occupational rehabilitation following injury
Source: PLoS One. 2025 Jul 11;20(7):e0327355. doi: 10.1371/journal.pone.0327355 (PMC12250564; doi:10.1371/journal.pone.0327355)
Supplement: S4 Table — (DOCX) [file pone.0327355.s004.docx]

**S4 Table:** Revised Beliefs and Perceptions items

| Allocated Positivum Beliefs and Perceptions Scales (PBPS) | ITEM | Likert scale response |
| --- | --- | --- |
| Health-related Work beliefs | I believe I am capable of working | Strongly agree (1) to Strongly disagree (5) |
| Work expectations | I am confident that I will be working / will still be working in 3 months | Strongly agree (1) to Strongly disagree (5) |
| Health-related Work beliefs | I believe my health will get worse while working | Strongly agree (1) to Strongly disagree (5) |
| Health-related Work beliefs | It is not really safe for me to work | Strongly agree (1) to Strongly disagree (5) |
| Employer Perceptions | Employers prefer not to hire people with disabilities | Strongly agree (1) to Strongly disagree (5) |
| Employer Perceptions | Because of my health, employers think that I am too much trouble | Strongly agree (1) to Strongly disagree (5) |
| Health-related Work beliefs | I should not work in my current condition | Strongly agree (1) to Strongly disagree (5) |
| Employer Perceptions | Employers worry that I will injure myself at work | Strongly agree (1) to Strongly disagree (5) |
| Health-related Work beliefs | I believe that my condition interferes with my ability to work | Strongly agree (1) to Strongly disagree (5) |
| Employer Perceptions | Employers worry that I will need too much time off work | Strongly agree (1) to Strongly disagree (5) |
| Health-related Work beliefs | I am concerned about my health and can’t think about work at the moment | Strongly agree (1) to Strongly disagree (5) |
| Perceived enjoyment of work | There are things I enjoy about working / think I would enjoy about working | Strongly agree (1) to Strongly disagree (5) |
