# Supplementary figures and images for: Psychometric evaluation of the Positivum beliefs and perceptions scales to inform occupational rehabilitation following injury
Source: PLoS One. 2025 Jul 11;20(7):e0327355. doi: 10.1371/journal.pone.0327355 (PMC12250564; doi:10.1371/journal.pone.0327355)

**S1 Fig**: **Scree plots of observed and random eigenvalues**

| 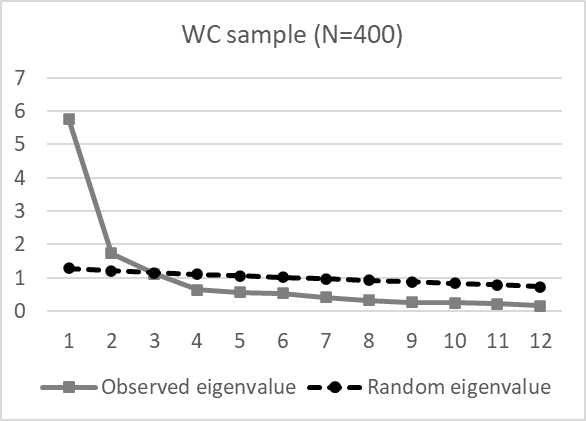 | 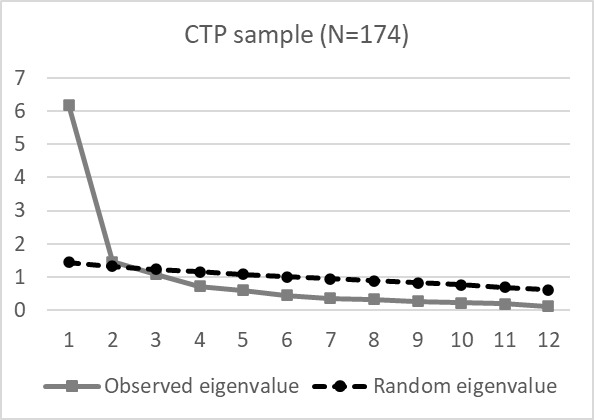 |
| --- | --- |

Supplement: S1 Fig — (DOCX) [file pone.0327355.s005.docx]
