# Supplementary material for: Psychometric evaluation of the Positivum beliefs and perceptions scales to inform occupational rehabilitation following injury
Source: PLoS One. 2025 Jul 11;20(7):e0327355. doi: 10.1371/journal.pone.0327355 (PMC12250564; doi:10.1371/journal.pone.0327355)
Supplement: S2 Fig — (DOCX) [file pone.0327355.s006.docx]

**S2 Fig**: **Category probability curves for Employer Perceptions items**

| **C/WC sample (n=400)** | |
| --- | --- |
| Item 5  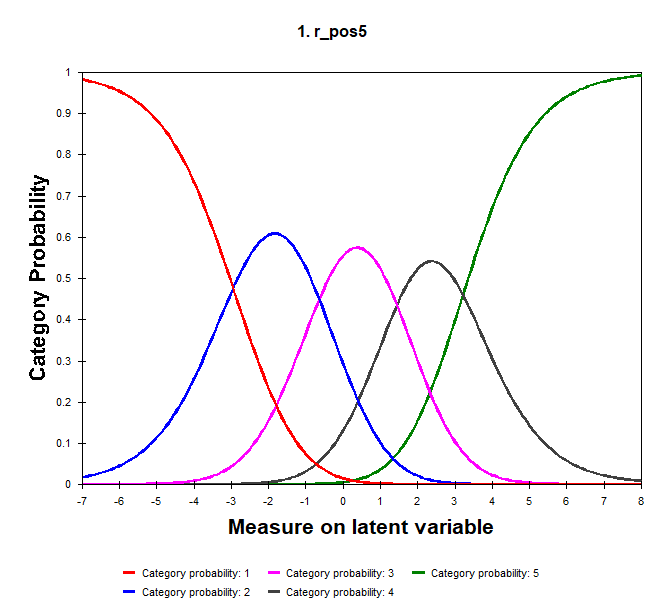 | Item 6  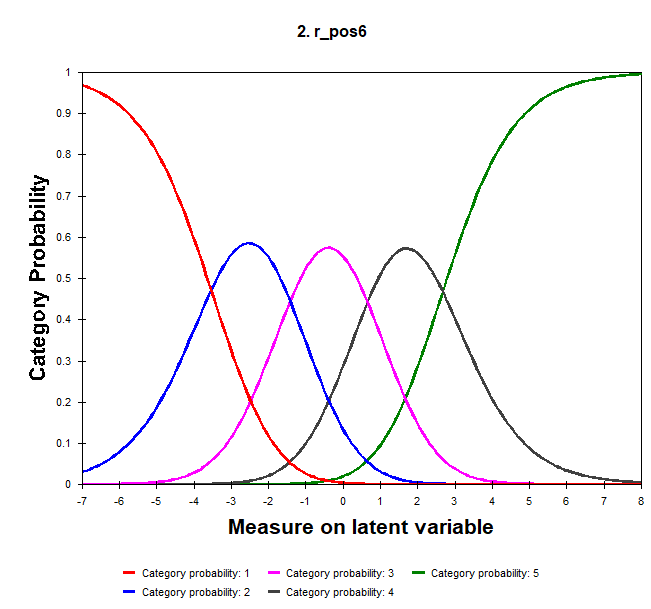 |
| Item 8  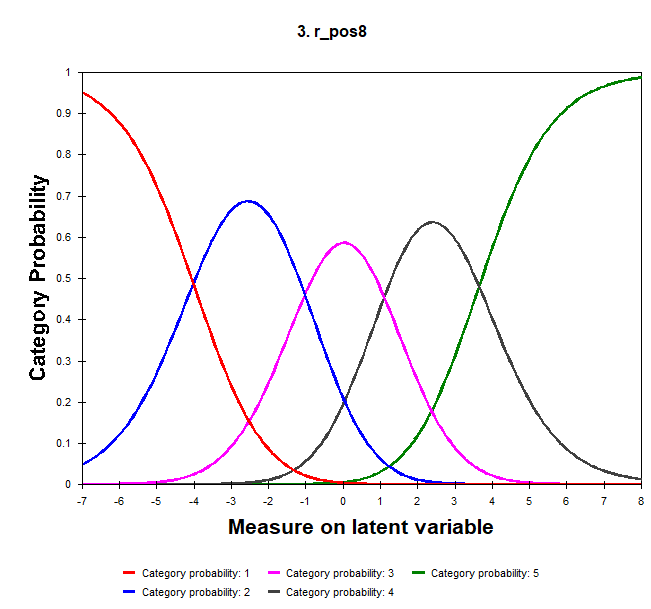 | Item 11  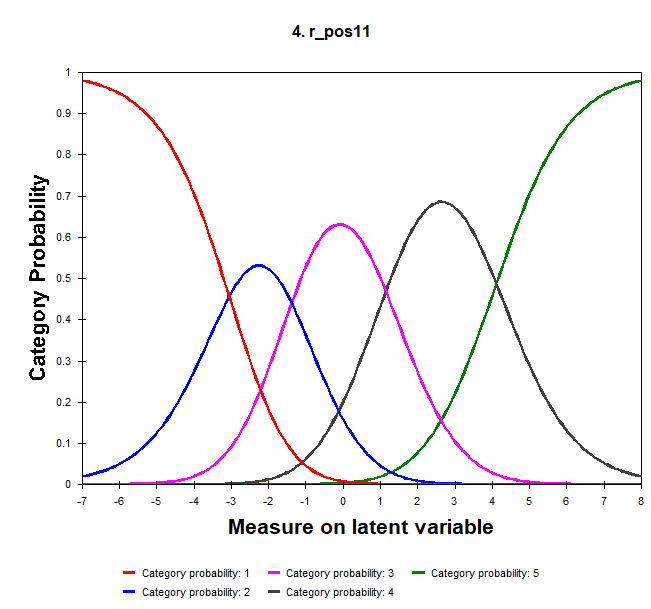 |
| **C/CTP sample (n=174)** | |
| Item 5  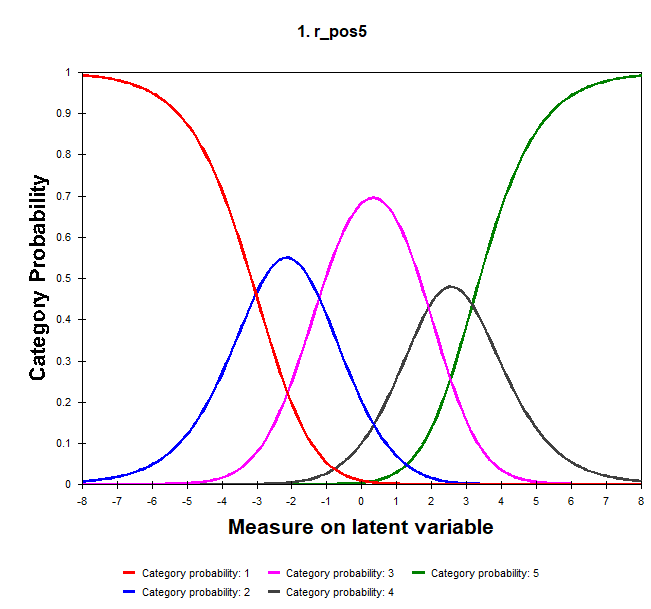 | Item 6  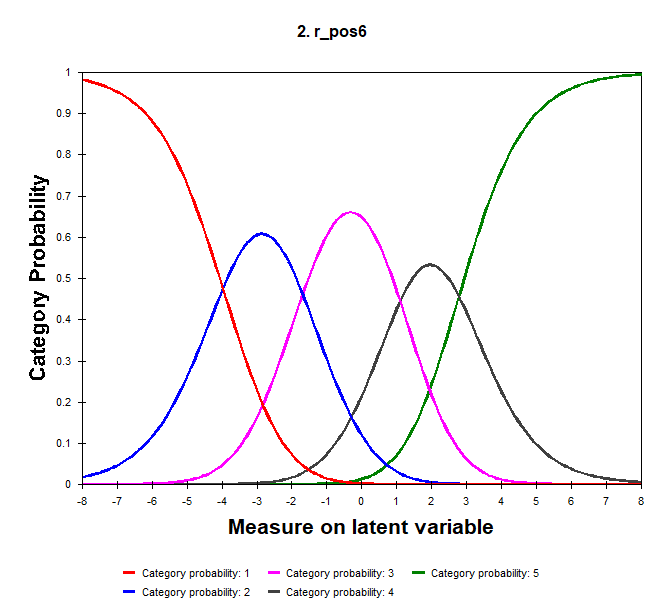 |
| Item 8  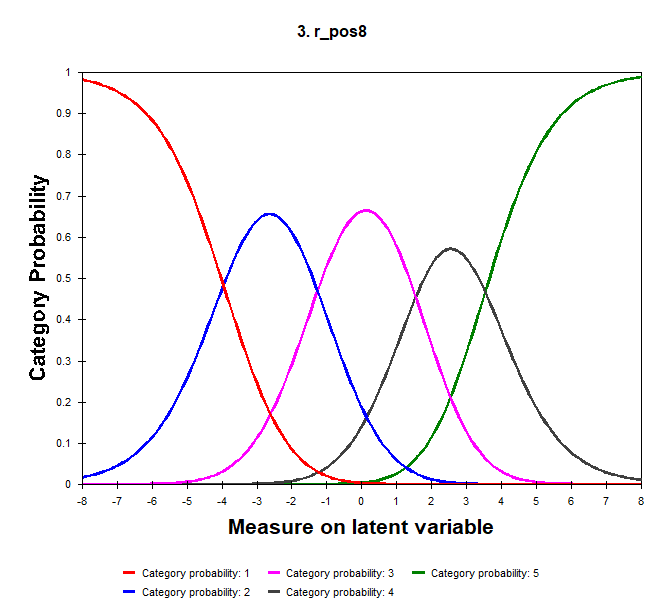 | Item 11  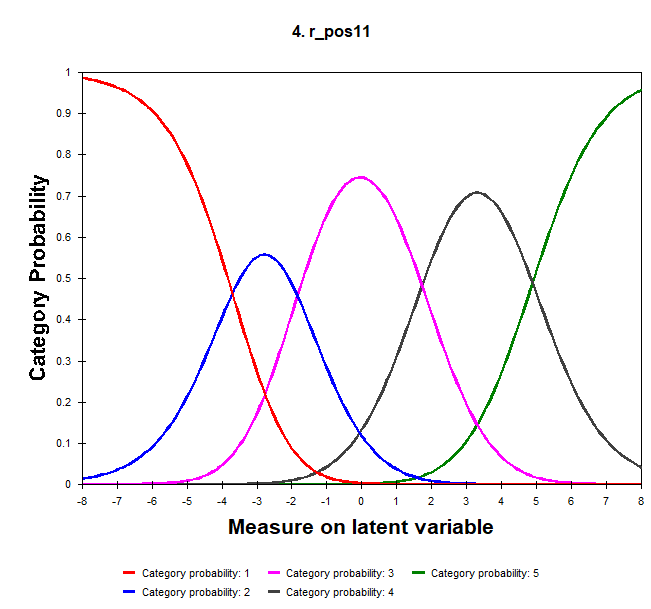 |

Abbreviations: C/WC = calibration sample, Workers Compensation scheme; C/CTP = calibration sample, Compulsory Third Party insurance scheme.
