# Supplementary material for: Psychometric evaluation of the Positivum beliefs and perceptions scales to inform occupational rehabilitation following injury
Source: PLoS One. 2025 Jul 11;20(7):e0327355. doi: 10.1371/journal.pone.0327355 (PMC12250564; doi:10.1371/journal.pone.0327355)
Supplement: S3 Fig — (DOCX) [file pone.0327355.s007.docx]

**S3 Fig**: **Category probability curves for Health-related Work Beliefs items**

| **C/WC sample (n=400)** | |
| --- | --- |
| Item 1  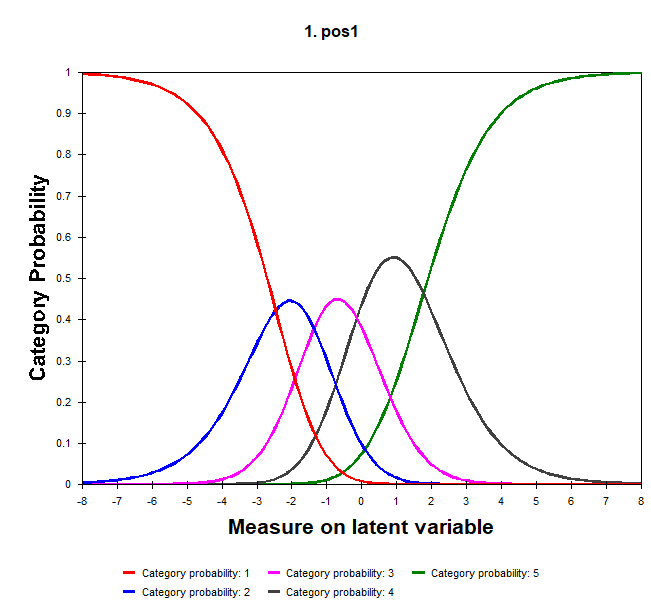 | Item 3  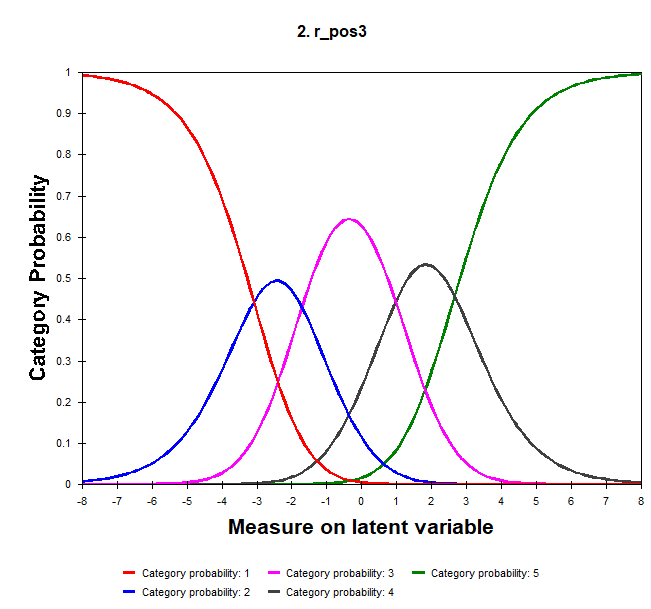 |
| Item 4  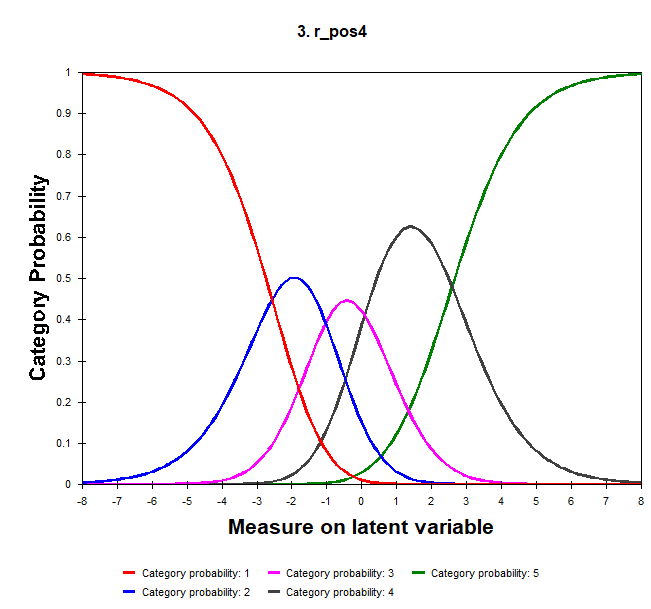 | Item 7  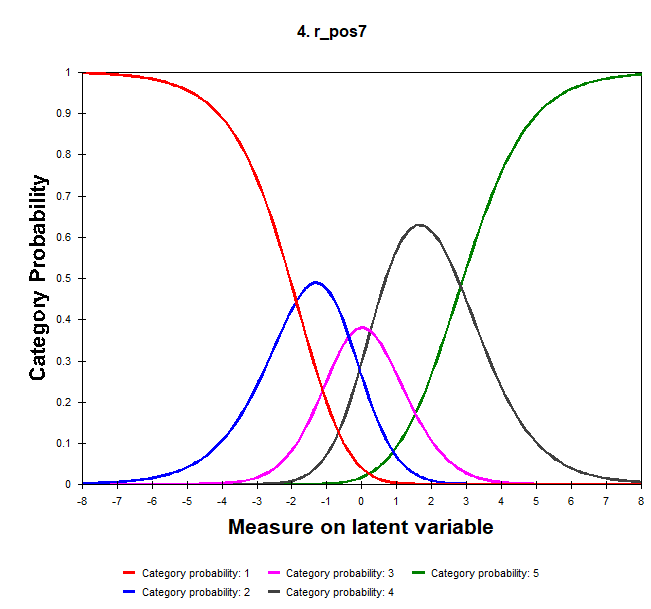 |
| Item 9  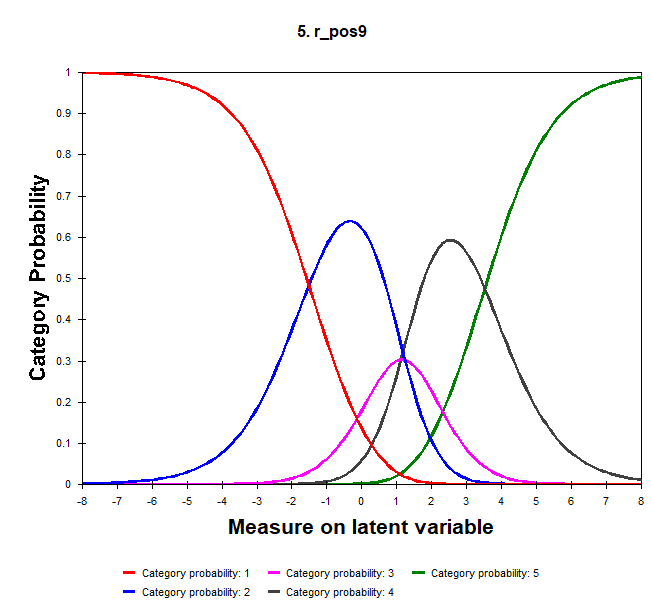 | Item 10  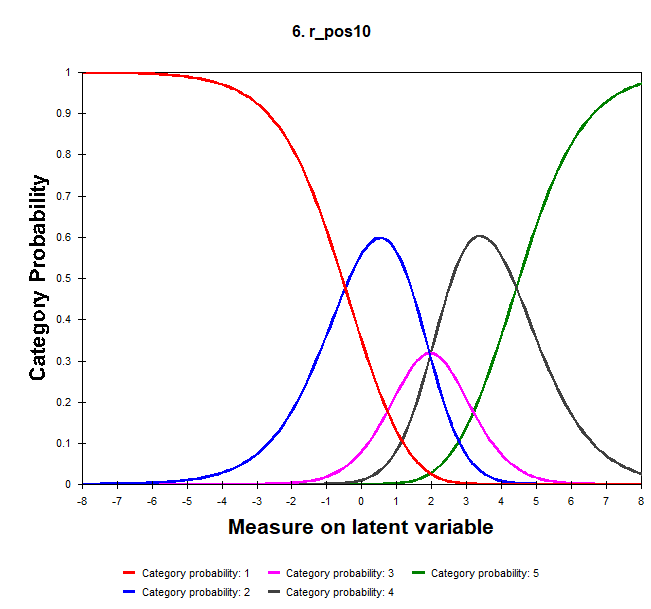 |
| Item 12  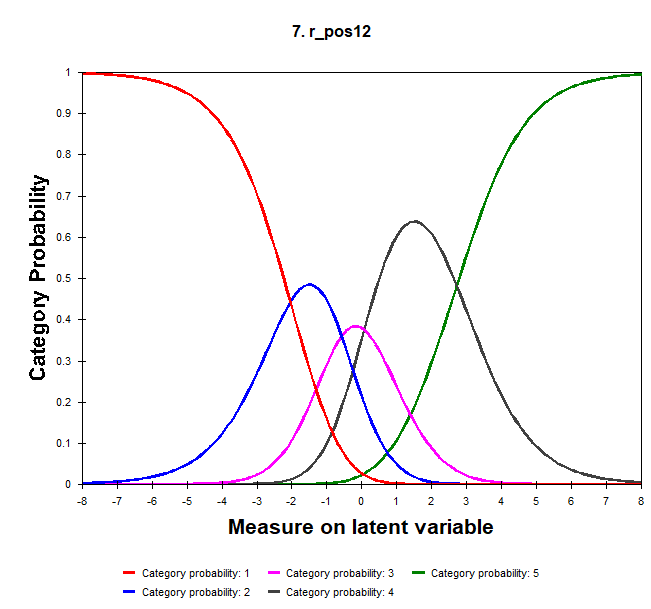 | Item 13  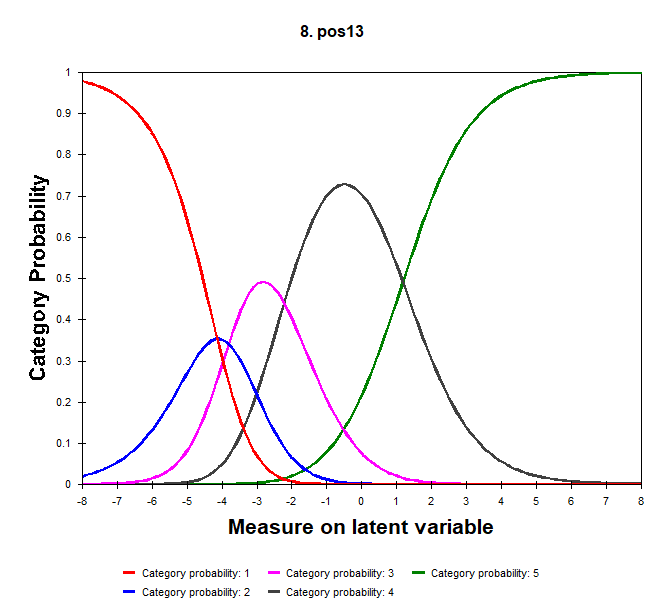 |

| **C/CTP sample (n=174)** | |
| --- | --- |
| Item 1  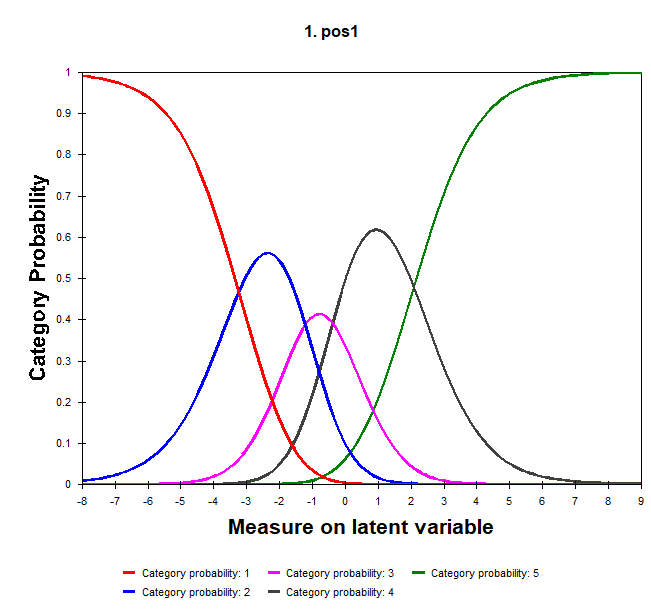 | Item 3  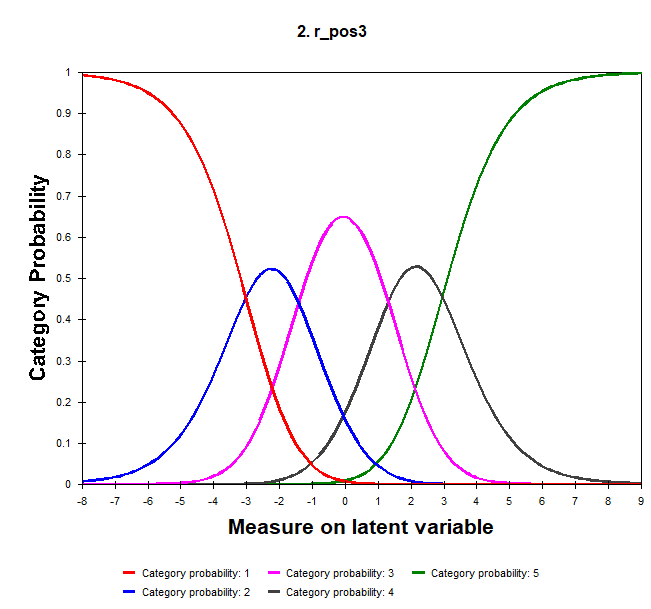 |
| Item 4  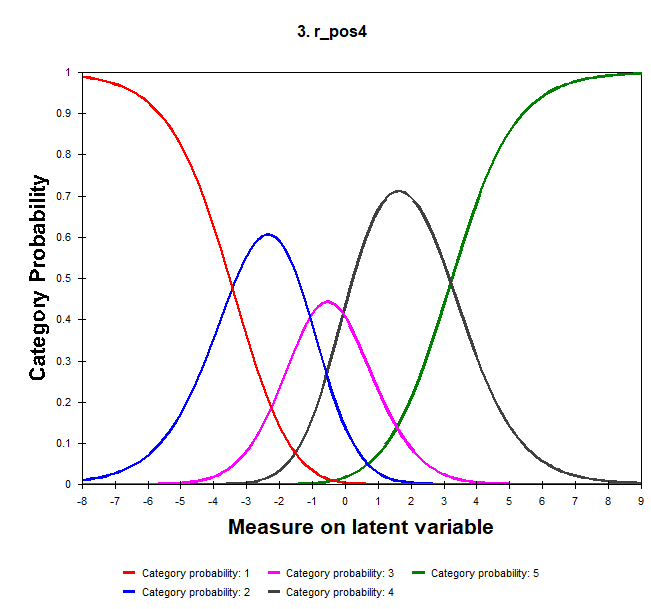 | Item 7  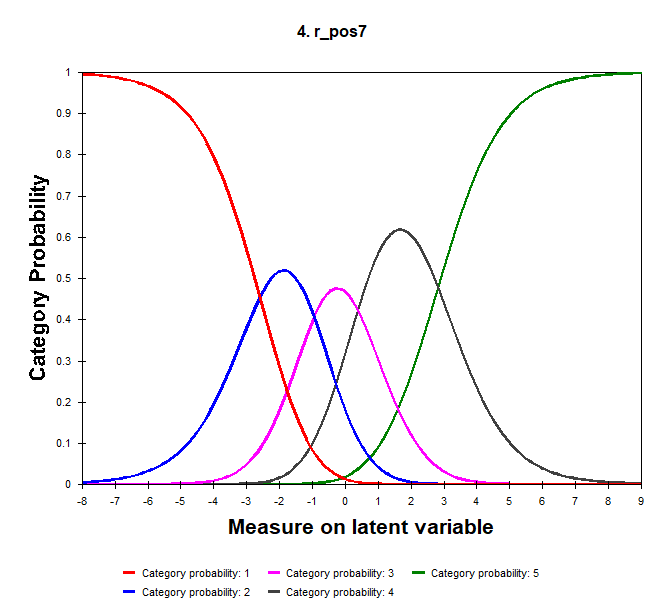 |
| Item 9  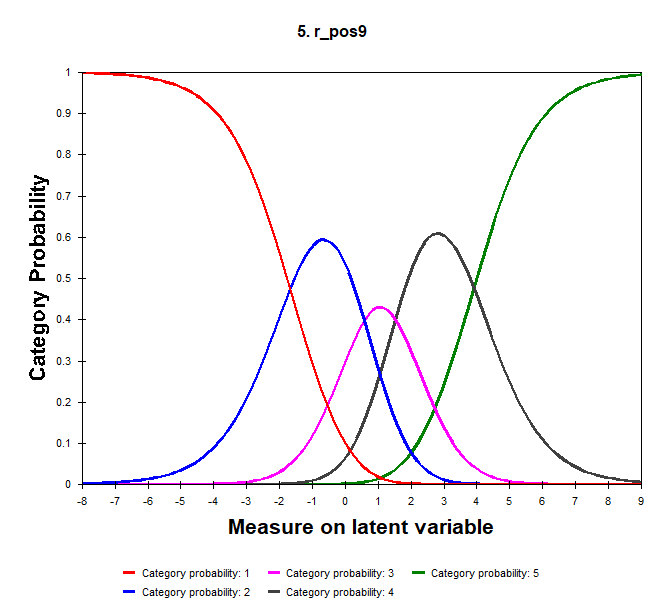 | Item 10  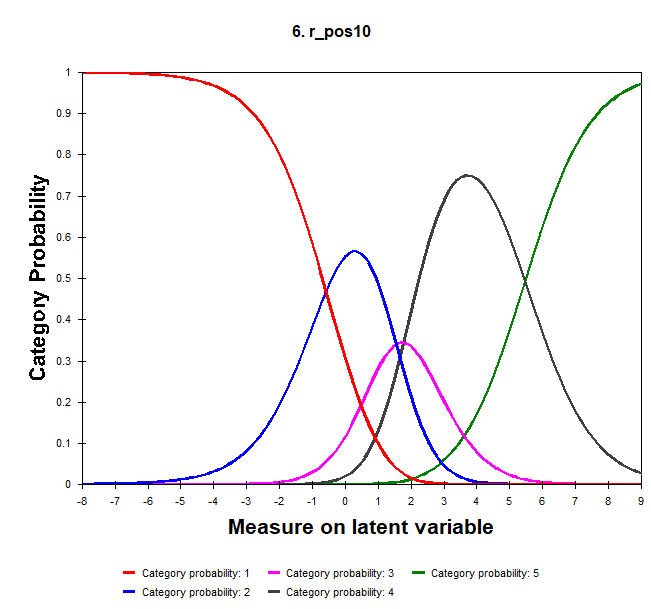 |
| Item 12  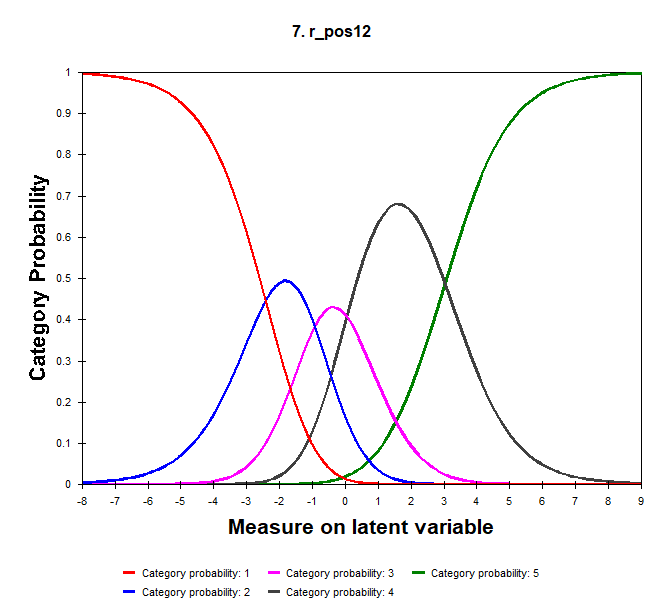 | Item 13  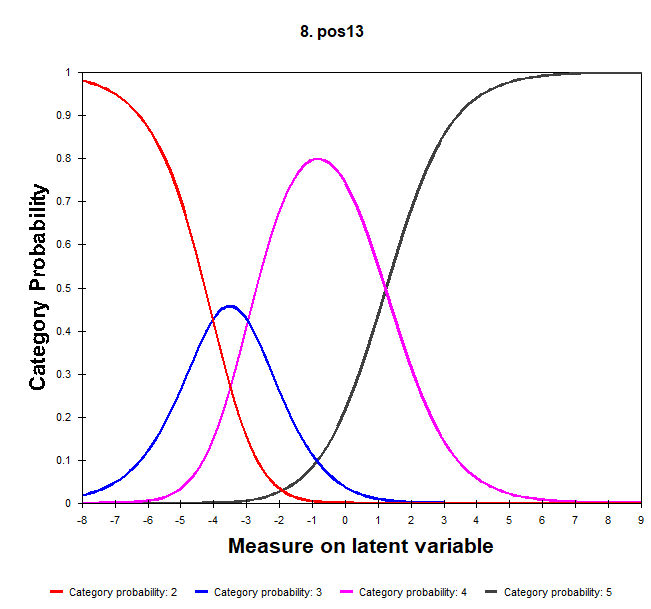 |

Abbreviations: C/WC = calibration sample, Workers Compensation scheme; C/CTP = calibration sample, Compulsory Third Party insurance scheme.
